# ‘Green’ Cr(iii)–glycine electrolyte for the production of FeCrNi coatings: electrodeposition mechanisms and role of by-products in terms of coating composition and microstructure†

**DOI:** 10.1039/c9ra04262h

**Published:** 2019-08-16

**Authors:** Enrico Bertero, Cristina V. Manzano, Eva Pellicer, Jordi Sort, Robert M. Ulfig, Stefano Mischler, Johann Michler, Laetitia Philippe

**Affiliations:** Empa – Swiss Federal Laboratories for Materials Science and Technology, Laboratory for Mechanics of Materials and Nanostructures Feuerwerkerstrasse 39 3602 Thun Switzerland enrico.bertero@empa.ch cristina.vicente@empa.ch johann.michler@empa.ch laetitia.philippe@empa.ch +41 58 765 69 90 +41 58 765 63 93; Ecole Polytechnique Fédérale de Lausanne, Tribology and Interfacial Chemistry Group, Materials Institute Station 12 (SCI-STI-SM) 1015 Lausanne Switzerland stefano.mischler@epfl.ch; Departament de Física, Facultat de Ciències, Universitat Autònoma de Barcelona 08193 Bellaterra Spain eva.pellicer@uab.cat jordi.sort@uab.cat; Institució Catalana de Recerca i Estudis Avançats (ICREA) Pg. Lluís Companys 23 08010 Barcelona Spain; CAMECA Instruments Inc. 5470 Nobel Drive Madison WI 53711 USA robert.ulfig@ametek.com

## Abstract

The electrodeposition of stainless steel-like FeCrNi alloys for miniaturised devices is appealing as it would allow combining excellent material properties (*e.g.* corrosion resistance, hardness, biocompatibility) at low-cost. However, conventional baths often contain hazardous hexavalent chromium. Cr-based alloys electrodeposited from environmentally friendly trivalent chromium electrolytes are crucial for industrial application for facilitating the transition towards sustainable and ecological production and processing. Nevertheless, this process has not been comprehensively studied or understood in depth: especially the role of organic agents (common additives for improving Cr(iii)-based plating; *e.g.* glycine) in terms of material properties of the electrodeposits. The aim of this work was to investigate the electrodeposition of FeCrNi coatings from a ‘green’ Cr(iii)–glycine electrolyte. Novel information was attained by analysing films developed under various conditions and characterising them using a combination of advanced techniques. The evolution of microstructure (from amorphous to nanocrystalline) in correlation with film composition (*i.e.* metals ratio and presence of impurities) and elemental 3D spatial distribution was achieved for coatings produced from different anode materials and thermal post-treatment. The influence of Cr(iii) and glycine in terms of coating atomic contents (*i.e.* Fe–Cr–Ni–O–C–N–H) was evaluated for films in which both the applied current density and electrolyte composition were varied. These results, together with a thorough analysis on metals speciation/complexation allowed us to propose various Cr(iii)-based electroreduction mechanisms, and to observe, upon annealing, segregation and distribution of impurities, as well as of oxides and metals with respect to microstructure variation, providing an explanation for the amorphisation process.

## Abstract 2

Electrodeposition mechanisms of a ‘green’ FeCrNi Cr(iii)–glycine electrolyte and their correlation with coatings' composition (metals/impurities), microstructure and elemental distribution variations.

## Graphical abstract

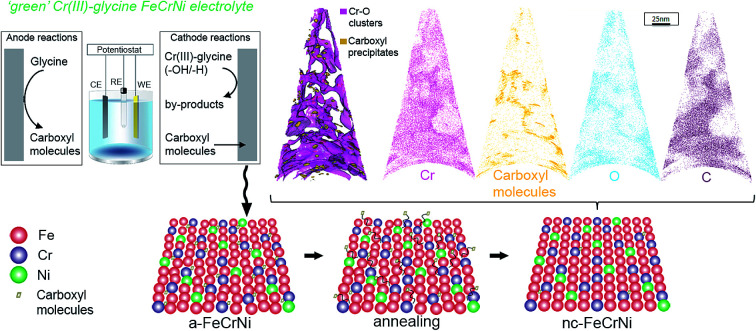

## 1. Introduction

Electrodeposition of chromium-based films has been widely used in various industrial sectors, due to several process advantages (*e.g.* versatility, scalability, cost-effectiveness) and many outstanding properties of Cr coatings (hardness, wear and corrosion resistance, *etc.*).^1^ However, the commonly used hexavalent chromium electrolyte must be replaced, as it is toxic and its waste poses an environmental threat. Conversely, the trivalent chromium plating electrolyte constitutes a ‘green’ and effective alternative to Cr(vi) baths.^2^ Unfortunately, Cr coatings obtained from trivalent chromium electrolytes do not yet match the outstanding characteristics of the films produced using Cr(vi) baths. The reactions taking place during trivalent chromium electrodeposition are very complex and have yet to be thoroughly investigated. Organic compounds are usually added to the electrolyte to form Cr(iii)-complex molecules, which improve the deposition process. However, they are a source of unwanted impurities, *e.g.* carbon. Many studies have focussed on Cr(iii) deposition mechanisms without investigating the pathways of how the main impurities are incorporated in the films and how they can affect the material properties of such coatings. Additionally, more complex electrolytes including other metallic ions (*e.g.* transition metals like Fe and Ni) are seldom mentioned in the literature. FeCrNi electrodeposits similar to austenitic stainless steel are of great interest for creating state-of-the-art micro-nano components, which could be used in the bio-medicine/micro-robotic sector. Therefore, a study of the mechanisms occurring in Cr(iii)-based electrolytes containing other metal ions and organic complexing agents is essential for the development of this important process.

The electroreduction mechanism of the trivalent chromium electrolyte was shown to follow a step-wise mechanism with the formation of rather stable intermediate Cr(ii) compounds:^3–6^

1 Cr(iii) → Cr(ii) *E*_0_ = −0.41 V *vs.* SHE

2 Cr(ii) → Cr(0) *E*_0_ = −0.91 V *vs.* SHE

Step (2) has been accepted to be the limiting reaction in the Cr(iii) to Cr(0) reduction process.^3^ The Cr(iii) ion forms chromium–aqua complexes, such as [Cr(H_2_O)_6_]^3+^, which are characterised by low kinetic activity due to the close-ordered hexa-water structure that prevents the central Cr^3+^ ion to exchange electrons with the cathode surface.^4,7,8^ For this reason, a complexing agent (*e.g.* glycine, formate, oxolate, acetate or citrate) is often introduced into the electrolyte to destabilise the chromium–aqua complex and enable chromium deposition. Additionally, pH buffers (*e.g.* boric acid) are added to hinder hydrolysis and olation (and, ultimately, polymerisation). When in close proximity to the cathode surface, chromium-complexes typically undergo these reactions when the pH has increased because of hydrogen evolution reaction (HER).^8^

Glycine (NH_2_CH_2_COOH) has been used in trivalent chromium deposition, as it is both a strong complexing agent and exhibits pH buffering properties, allowing to avoid olation reactions.^7^ McDougall *et al.*^9^ stated that optimal deposition results (*i.e.* high deposition rate, good surface morphology and uniformity) could be obtained from an electrolyte with Cr(iii) : glycine molar ratio of 1 : 1, resulting in monoligand Cr(iii)–glycine as the main compound present inside the electrolyte. Still the electroreduction mechanism involved are not completely understood, in fact, the vast majority of works using complexed chromium electroplating (*e.g.* formate,^4,5^ acetate,^5^ glycine^7,9^) propose that the complexing ligands are completely released during the reduction process (and stabilised by cations present in the electrolyte). However, in many studies^10–12^ the presence of impurities (mainly carbon) and the formation of compounds, such as carbides, provide evidence of the participation of these organic molecules and the incorporation of their moieties during Cr(iii)-complex deposition process.

The purpose of electrodepositing multicomponent alloys containing Cr is to obtain a combination of properties that cannot be achieved by pure Cr coatings. For example, ferromagnetic elements such as Fe, Ni and Co are utilised to confer a magnetic response to the electrodeposited films.^13,14^ In general, magnetic, corrosion and mechanical properties of electrodeposited coatings are dictated by the amount and nature of the transition metals and alloying elements.^15^ Biocompatibility as a material property can be obtained from very few electrodeposited materials, mainly CrCo and stainless steel such as FeCrNi-based alloys.^16,17^

The use of Fe and Ni in a Cr(iii)-complex electrochemical system can be found in the literature for FeNiCr^20–24^ and FeNiCrMo^25^ electrodepositions. However, these works mainly focus on the influence of deposition parameters on film features, overlooking the role of the electrochemical reactions on the coating's composition and properties. Only one of our previous publications^26^ tackled the electrodeposition of FeCrNi from a Cr(iii)–glycine electrolyte and put forward an explanation for the source of impurities incorporated in the films. According to this study, the use of an inert platinum anode was the cause of the oxidation of glycine, which resulted in the formation of by-products (*e.g.* formaldehyde, formic acid). These were, in turn, the main source for carbon inclusion and consequent amorphisation of the deposit. By obtaining nanocrystalline low-carbon (0.6 wt%) FeCrNi coatings on a double-cell set-up with separated anode and cathode compartments, it was thought that the amorphisation mechanism was caused by inclusion of interstitial carbon, present in a supersaturated state. Nonetheless, that preliminary work did not propose an electrochemical pathway for carbon incorporation. Additionally, the fact that carbides were not detected differs from many similar studies on Cr(iii)–formate electrolytes.^10–12^ Only Protsenko *et al.*^11^ suggested a possible mechanism for carbon inclusion from a trivalent chromium bath containing formic acid as the complexing agent.

On one hand, the presence of carbides increases the hardness of stainless steels.^27^ On the other, high amounts of carbon/carbides are undesirable as they can decrease corrosion resistance and, therefore, the biocompatibility of Cr-based coatings.^28^ Other impurities, such as nitrogen and hydrogen are also known to have an impact on the morphology and properties of alloys containing chromium like stainless steels.^27^ According to Gabe,^29^ a local rise of pH at the cathode results in the formation, adsorption and incorporation of Cr hydrides (Cr–H). H incorporation is thought to play a key role in the coating's porosity and embrittlement, lowering deposition efficiency and producing amorphous films.

There is a lack of thorough research conducted to explain or to better comprehend the possible mechanisms involved in complexed Cr(iii)-based alloys electroplating and how they are linked to the obtained films' characteristics. This is particularly true regarding the pathways by which impurities are incorporated in the deposits, as they are as important as the main metallic elements in determining the coatings' material properties. It is crucial to investigate how the morphology, microstructure and film composition are affected by deposition parameters and post-processing for this systems. This is a necessary step for further improving Cr(iii) electrodeposition, considering that the process is of great importance for environmentally friendly and sustainable Cr-based applications.

The objective of this work is to further investigate the considered system consisting of Cr(iii)–glycine complexes, Fe and Ni ions, in terms of morphology, microstructure and composition of the final electrodeposits.

The influence of impurities and deposition parameters on such material's properties is achieved by first varying the anode material and then the electrolyte composition, in combination with post-treatments (*i.e.* thermal annealing) and several advanced characterisation techniques. In particular, X-ray diffraction (XRD) and X-ray photoelectron spectroscopy (XPS) are utilised to obtain information on the correlation between coating microstructure and composition, while taking into account the chemical states of Fe, Cr, Ni, O, C and N. Additionally, He elastic recoil detection analysis (He ERDA) is used to accurately determine the hydrogen content throughout the coating's thickness. Furthermore, 3D atom-by-atom elemental reconstruction is implemented using cutting-edge atom probe tomography (APT). This XPS complementary technique is used to precisely determine which elements/compounds are present and how they are spatially distributed within the FeCrNi coatings at the nano-scale, providing information regarding material inhomogeneity (*i.e.* clustering, phase separation, precipitates) and coating elemental evolution during microstructural and material composition variations (before and after annealing process). The influence of glycine in terms of metals complexation and information on Cr oxidation species within different Cr-based electrolytes were evaluated by means of chemical equilibrium diagrams and UV-vis spectra, respectively.

Based on all of the obtained results, some mechanisms are proposed to explain the following processes involved in the amorphisation of the studied FeCrNi electrodeposits:

(1) Complexed Cr(iii) electroreduction.

(2) Complexing agent (*i.e.* glycine) side-reactions.

(3) Influence of transition metals (*i.e.* Fe, Ni) on cathodic reduction processes.

## 2. Experimental methods

FeCrNi films were deposited using a standard three electrode electrochemical cell equipped with a water jacket for temperature control. The temperature of the electroplating bath was set to 22 °C ± 0.5 °C using a temperature controlled circulator (Julabo, F12-ED). Portions of silicon wafer covered with a sputter-coated Au layer (100 nm) deposited on top of a Cr adhesion layer (5 nm) were used as substrates. Prior to each electrodeposition experiment, the substrate was cleaned in a freshly prepared Piranha solution (30% H_2_O_2_ : H_2_SO_4_ = 1 : 3) and thoroughly rinsed in deionized water (18.2 MΩ cm). Subsequently, the substrate was partially masked with plastic tape to expose a plating area of 1.5 cm × 1.5 cm. A saturated Ag/AgCl electrode was used as the reference electrode. Depending on the experiment, a Pt mesh (80 mesh, 25 mm × 35 mm, ALS Co., Ltd) or pure Ni pellets inside a platinised titanium basket (PtTi fine mesh, *H* × *W* × *D*: 65 mm × 50 mm × 15 mm) were used as a counter-electrode. The bath composition for FeCrNi electrodeposition is shown in Table 1. This electrolyte composition was found to be the most adequate for obtaining coatings with the right balance of Fe–Cr–Ni similarly to metallurgical austenitic stainless steel (18Cr–11Ni).^30^ The difference in concentration between Fe and Ni salts is due to the so-called anomalous co-deposition, which favours Fe electroreduction. High amounts of chromium salt are necessary, as a large reduction potential is needed for the deposition of this metal.

**Table 1:** Bath composition for FeCrNi electrodeposition^a^

Components	Concentration (mol L^−1^)
CrCl_3_·6H_2_O	0.4
Glycine (NH_2_CH_2_COOH)	0.4
FeCl_2_·4H_2_O	0.03
NiCl_2_·6H_2_O	0.2
NH_4_Cl	0.5
H_3_BO_3_	0.15
NaCl	0.5

a pH: 1, temperature: 22 °C.

All the chemicals were of reagent grade (Sigma-Aldrich) and were used as-received without any further purification. The bath constituents were mixed by following the procedure described by Hasegawa *et al.*^26^. The Cr(iii) and glycine solution was first complexed at 80 °C for 30 minutes, to which a second solution, comprising of all the other elements, was added. The electrodeposition was carried out in galvanostatic mode using a potentiostat (PGSTAT 302N, Metrohm Autolab B.V.) controlled by NOVA (version 2.1) software. The used electrolytes were aged galvanostatically for a few hours at a constant current density to obtain films with good surface morphology and uniformity.

Two investigation methods were performed to better understand the deposition mechanisms involved in the studied Cr(iii)-based FeCrNi electrolyte. Particular emphasis was placed on the role of Cr(iii), additives (*i.e.* glycine) and ferromagnetic elements (*i.e.* Fe and Ni) on material composition and microstructural changes of FeCrNi electroplated films.

The first method involved studying the influence of the anode material by electrodepositing FeCrNi alloys using a platinum anode (*Pt anode*) and a pure nickel anode (*Ni anode*). The applied deposition parameters were the same for both cases (current density = −80 mA cm^−2^, total deposited charge density ≈ 200 C cm^−2^) resulting in average deposition potentials of −1.44 V *vs.* Ag/AgCl and −1.41 V *vs.* Ag/AgCl for the *Pt anode* and *Ni anode* cases, respectively. Next, the samples were annealed using a rapid thermal annealing (RTA) system (MILA-5050, ULVAC) under an Ar controlled atmosphere (99.9999% purity) for 1 hour at 600 °C (ramp up and ramp down of 10 °C min^−1^).

In the second method, the influence of Cr(iii)–glycine complexation was determined by using three different electrolytes: *Standard* (as described by Table 1), *No Cr–Glycine* (*Standard* without Cr and glycine) and *No Cr* (*Standard* without Cr). FeCrNi electrodeposition was performed galvanostatically at −60 and −80 mA cm^−2^ (total deposited charge density ≈ 50 C cm^−2^) with the *Standard* electrolyte resulting in average deposition potentials of −1.30 V *vs.* Ag/AgCl and −1.44 V *vs.* Ag/AgCl, respectively. For the *No Cr–Glycine* and *No Cr* baths electrodeposition at current density of −80 mA cm^−2^, average deposition potentials were −1.55 V *vs.* Ag/AgCl and −1.39 V *vs.* Ag/AgCl, respectively. A Pt mesh was used as the counter electrode in all experimental variations.

The surface morphology of the FeCrNi samples was observed *via* field-emission scanning electron microscopy (FE-SEM, Hitachi S-4800, Hitachi High-Technologies Corporation). Crystal structures were characterised by means of *in situ* Bragg–Brentano X-ray diffraction (BB-XRD, Brucker) with a Cu Kα radiation source (40 kV, 40 mA). Diffractograms were recorded in the 2*θ* range between 15° and 100°. An offset of 1° was used to avoid the signal from the Si substrate. Moreover, possible fluorescence signals associated with the presence of Fe in the films were filtered by using a larger lower discriminator. The mean size of the crystallite *τ* (more specifically, the coherent diffraction length) was calculated using the Debye–Scherer equation:
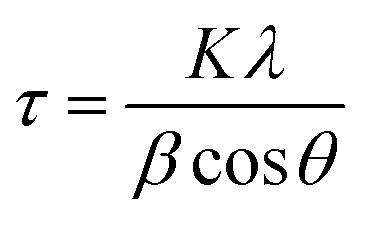
where: *K* is the shape factor (0.9), *λ* the radiation wavelength, *β* is the broadening of full width half maximum (FWHM) of the selected peak and *θ* the corresponding Bragg angle. The used peaks and Bragg angles were (110) peak at 2*θ* = 45° for the α-Fe phase and (111) peak at 2*θ* = 44° for the γ-Fe phase. Fitting of the XRD peaks was performed by using the Crystallographic Open Database – COD.^31^

X-ray fluorescence (XRF, Fischerscope® X-RAY XDV®-SDD, Fischer Technology) was used to estimate the Fe–Cr–Ni wt% and thicknesses along the sample area. The measurements were performed at 25 uniformly distributed points on each specimen. The film was characterised by an uneven composition from the edges to the centre of the sample (Cr variation ≈15 wt%), caused by the primary current distribution during electrodeposition. Therefore, the averages of 9-matrix central points on each sample were chosen as representative concentrations. Approximate current efficiencies (C.E., %) from XRF thickness measurements were calculated by knowing the weight percentage of each metal element, assuming the density of stainless steel to be *ρ*_SS_ = 7.93 g cm^−3^ and using Faraday's law.

The elemental composition (at%) and chemical binding energies (BE) of the metal species (Fe, Cr and Ni) and impurities (O, C and N) were evaluated *via* X-ray photoelectron spectroscopy (XPS) on the coatings' surface and profiling at different depths from the surface down to approximately 500 nm. This was achieved by sputtering the material with Ar^+^ ions (2 or 4 keV sputtering energy). XPS data was acquired using various equipment: Physical Electronics (PHI) 5500 Multi-technique System‡‡Monochromatic Al Kα X-ray radiation source (*hν* = 1486.6 eV) of 350 W power with a typical beam diameter of 800 μm. Hemispherical capacitor electron-energy analyser equipped with a multichannel plate detector. Electron take-off angle of 45° and analyser operated in the constant pass energy mode at 23.50 eV. Compensation of eventual surface charging with built-in electron neutralizer. Base pressure of the system below 1 × 10^−8^ Pa. Binding energy calibrated using Ag 3d_5/2_ at 368.21 eV with FWHM of 0.8 eV. (to investigate the influence of anode type on as-deposited samples), Physical Electronics (PHI) Quantum 2000 Scanning ESCA Microprobe System§§Monochromatic Al Kα X-ray radiation source (*hν* = 1486.7 eV) of 29.7 W power with a typical beam diameter of 150 μm. Hemispherical capacitor electron-energy analyser equipped with a channel plate and a position-sensitive detector. Electron take-off angle of 45° and analyser operated in the constant pass energy mode at 29.35 eV. Compensation of eventual surface charging with built-in electron and argon ion neutralizers. Base pressure of the system below 5 × 10^−7^ Pa. Binding energy calibrated using Cu 2p_3/2_, Ag 3d_5/2_ and Au 4f_7/2_ at 932.62 eV, 368.21 eV and 83.96 eV, respectively to within ±0.1 eV. (to investigate the effect of thermal annealing) and Physical Electronics (PHI) VersaProbe II Scanning XPS Microprobe System¶¶Monochromatic Al Kα X-ray radiation source (*hν* = 1486.7 eV) of 25.2 W power with a beam diameter of 100 μm. Spherical capacitor electron-energy analyser set at 45° take-off angle respect to sample surface. Analyser operated in the constant pass energy mode at 46.95 eV. Compensation of eventual surface charging with built-in electron and argon ion neutralizers. Base pressure of the system below 5 × 10^−7^ Pa. Binding energy calibrated using Cu 2p_3/2_, Ag 3d_5/2_ and Au 4f_7/2_ at 932.62 eV, 368.21 eV and 83.96 eV, respectively to within ±0.1 eV. (to determine the impact of Cr(iii)–glycine complexation on deposit properties by varying the electrolyte composition).

XPS spectra were analysed using CasaXPS software^32^ (version 2.3.19). The spectra were charge corrected by shifting all binding energies (BE) with respect to adventitious carbon (C 1s; C–C, C–H) at 284.8 eV. The regions of interest and corresponding relative sensitivity factors (R.S.F.) for elemental quantification and fitting were 1s for oxygen, carbon and nitrogen, and 2p_3/2_ for Fe, Cr and Ni elements. Core level XPS peak fitting was performed using an asymmetric line shape defined in CasaXPS as LF(*α*, *β*, *w*, *m*), where *α* and *β* set the spread of the tail on each side of the Lorentzian component. The *w* parameter is the damping factor, which gives the integration limit for tail reduction and *m* is the width of the Gaussian convoluted with the Lorentzian. All other components were fitted using a Gaussian (*Y*%)–Lorentzian (*X*%) profile defined in CasaXPS as GL (*X*), where *Y* = 100 − *X* and the *X* value was varied depending on the analysed element (from 25 to 50). Moreover, a standard Shirley background was used for all spectra.

Additionally, H ratio in at% with respect to all metal species (Fe, Cr and Ni) was determined at depths up to 200 nm by helium elastic recoil detection analysis (He-ERDA, ETH Zurich) using a 2 MeV He beam and the absorber foil technique.^33^

Atom probe tomography (APT, EIKOS X,^34^ CAMECA Instruments Inc. – Madison – USA) was performed on the FeCrNi electrodeposited films. Lift-out specimens attached to W posts were sharpened into tip geometry with radius of ∼50 nm using focused ion beam annular milling. Atom probe analysis was conducted in laser pulsing mode with wavelength of 532 nm and laser pulse energy of 13–20 nJ operating at 200 kHz with a specimen base temperature of 50 K and detection rate set to 0.005–0.02 atom per pulse. The typical dataset size of each analysis is around 10–50 M ions. IVAS 3.8 software from CAMECA was used for data reconstruction, detailed analysis for local chemical identification/composition (deduced from mass-over-charge ratio) and distribution of impurities.

Chemical equilibrium diagrams and concentration of species were evaluated using Hydra-Medusa software.^35^ Additionally, UV-vis absorbance spectra were measured in the wavelength range between 300 to 700 nm using a UV-vis spectrophotometer (UV-vis Lambda 900 UV, PerkinElmer) for different electrolytes: freshly prepared and galvanostatically aged (using Pt and Ni anodes) FeCrNi solutions diluted 10 times in deionised water, trivalent decorative chromium and hard hexavalent chromium commercial electrolytes (riag Oberflächentechnik AG, Switzerland) diluted in deionised water 10 and 20 K times, respectively.

## 3. Results

### 3.1. Anode role investigation

#### 3.1.1. Surface morphology

The electrodeposit from a nickel anode (*Ni anode*) differs slightly in terms of appearance from that obtained using a platinum anode (*Pt anode*). Semi-bright grey coatings were achieved from both anodes, however the one produced using a nickel anode is less uniform, as the borders appear to be duller and more fragile. The differences between the samples are more pronounced after annealing: blue-violet semi-bright coloured surface from the *Pt anode* sample and dull-grey from the *Ni anode* one (pictures of the samples in ESI†). The colour variation from grey to blue could be caused by a change in the thickness of the passive oxide layer, based on stainless steel tempering colours and oxide growth theory.^27^

**Fig. 1:**
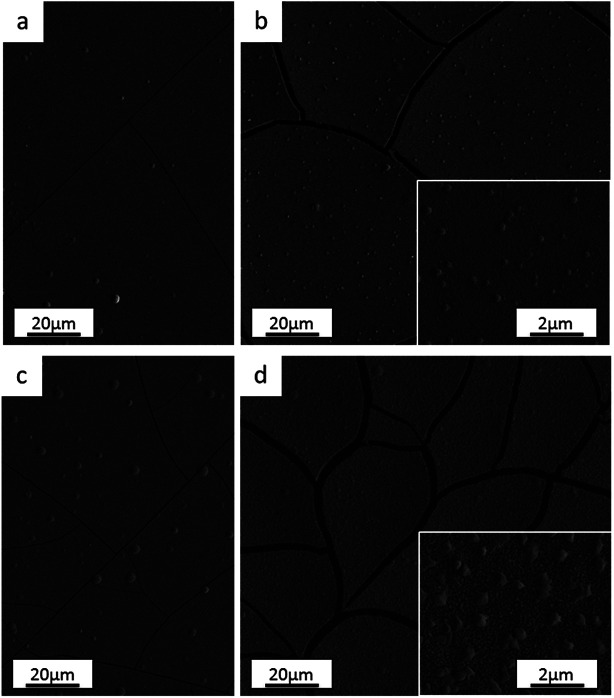
Surface morphology of electrodeposited FeCrNi films: *Pt anode* case (a) as-deposited and (b) after thermal treatment, *Ni anode* case (c) as-deposited and (d) after annealing process.

Optical microscopy and SEM observations show that the *Ni anode* sample (Fig. 1c) has more cracks than the *Pt anode* coating (Fig. 1a). As reported in previous similar works,^26,30^ the presence of cracks is mainly dependant on the thickness of the film (crack-free samples obtained when coating thickness is less than 5 μm). However, crack-free coatings (thickness ≤ 5 μm) started to crack immediately or a few days after deposition, when the Cr content in the coating exceeded a certain amount (Cr ≥ 28 wt%). This behaviour seems consistent with the incorporation mechanism of Cr hydrides within Cr(iii)-based electrodeposited alloys.^29^ High overpotentials (therefore increase in pH) result in the formation and incorporation of Cr hydrides (hexagonal close-packed, hcp), which are unstable and decompose into metallic Cr (body-centered cubic, bcc). This crystal phase transformation leads to approx. 15% volume shrinkage and, in turn, crack formation.

The annealed samples confirm the above-mentioned mechanism. In fact, SEM observations for both the *Pt anode* (Fig. 1b) and *Ni anode* (Fig. 1d) thermally treated samples show larger cracks with respect to the as-deposited counterparts. At high temperatures, unstable Cr hydrides easily decompose to metallic Cr, therefore producing more cracks. The FeCrNi electrodeposits present nodule-like features, which are undistinguishable in terms of composition with respect to the smooth surface, as observed in our previous work.^30^

Nevertheless, the difference in anode material does not particularly affect the surface morphology of the studied coatings.

#### 3.1.2. Crystal structure

**Fig. 2:**
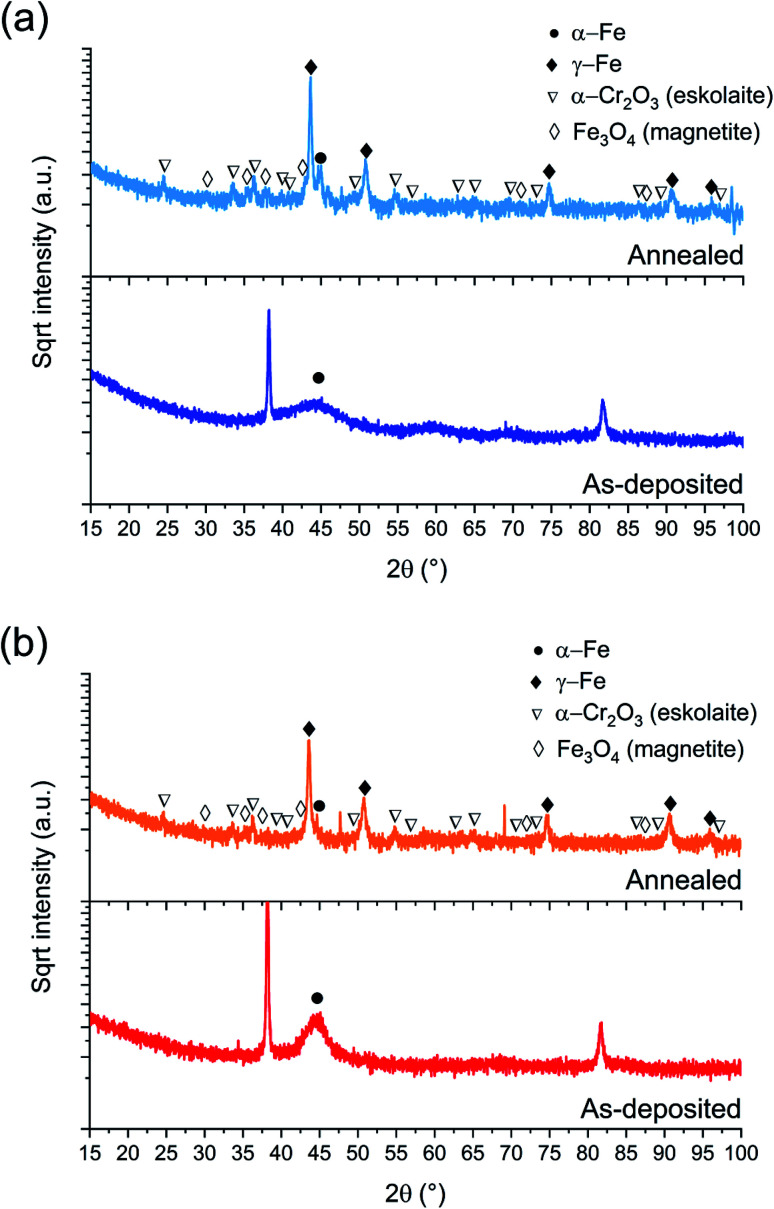
BB-XRD measurements of FeCrNi as-deposited and annealed (Ar atmosphere, 1 h at 600 °C) electrodeposits for the (a) *Pt anode* and (b) *Ni anode* coatings.

*Ex situ* Bragg–Brentano XRD measurements of the electrodeposited FeCrNi *Pt anode* (Fig. 2a) and *Ni anode* (Fig. 2b) films are shown in Fig. 2, both as-deposited and annealed under an Ar controlled atmosphere. Diffraction peaks at 2*θ* ≈ 38°, 69° and 82° correspond to face-centered cubic fcc Au (111), Si (100) and fcc Au (222) crystal planes (substrate contributions), respectively. The XRD diffractogram for the as-deposited *Pt anode* coating presents a very broad 2*θ* peak at approx. 45°, which highlights that the amorphous state has a tendency towards (110) reflection of the bcc α-Fe structure. In contrast, the as-deposited *Ni anode* film shows a narrower peak in the same position as previously stated, revealing an ultrafine-grained α-Fe material with a calculated crystallite size of approx. 5 nm.

The annealed samples (*Ni anode* and *Pt anode*) possess similar diffractograms. However, there is a clear difference between the as-deposited and annealed films. The thermally treated samples are characterised by many new peaks at approx. 44°, 51°, 75°, 91° and 95° corresponding to the (111), (200), (220), (113) and (222) crystal planes, respectively, which are reflections of the γ-Fe fcc phase. The mean crystallite size calculated from the most intense γ-Fe peak is in both cases in the range of 20–25 nm. Several small peaks are discernible in the diffractograms corresponding to iron oxides^36^ (Fe_3_O_4_ – magnetite) and chromium oxide (α-Cr_2_O_3_ – eskolaite). Moreover, in the case of the *Pt anode* annealed coating, although the predominant crystalline structure is γ-Fe, a distinct α-Fe peak is also visible at 45°, therefore showing that the film is a mixture of γ-Fe and α-Fe phases. The *Ni anode* annealed sample, in the other hand, does not exhibit a marked α-Fe contribution. It is worth mentioning that all diffractograms present a high background at low angles, which could be attributed to the presence of amorphous oxides.

It could be expected that the higher amount of Ni (see Section 3.1.3) in the *Ni anode* samples would cause γ-Fe to be the predominant phase in the electrodeposited films (nickel retains austenitic phase in stainless steel^27^). However, from one of our previous studies,^30^ the microstructure of FeCrNi films was found not to be influenced by Cr and Ni concentrations. Therefore, the microstructure diversity is anode-dependant and most probably related to impurity variations.

#### 3.1.3. Elemental composition, oxidation states and 3D atom-by-atom reconstruction

XRF was used to estimate the composition in wt% and current efficiency of the FeCrNi electrodeposited coatings. When a platinum anode was used (*Pt anode* case), the composition of the resulting coating was Fe57–Cr28–Ni15 (6.2% current efficiency, C.E.), whereas Fe52–Cr26–Ni22 (C.E. 8.3%) films were obtained from using a nickel anode (*Ni anode* case). The higher nickel content in the latter may be caused by higher amounts of nickel ions being released in the electrolyte during anode oxidation throughout the deposition process.

The elemental compositions in at% for all samples (*Pt anode*, *Ni anode*: as-deposited, annealed) including both metallic species (Fe, Cr and Ni) and impurities (O, C and N) were evaluated by means of XPS and are depicted in Table 2, both outer surface and in-depth (achieved by sputtering for overall 50 min) of the samples. In the same table, H content ratios with respect to the total metal species obtained from He-ERDA are also listed (the content inside the coating was determined after 20 min of Ar sputtering).

**Table 2:** Elemental composition of Fe–Cr–Ni–O–C–N (XPS) and of H (He-ERDA) of as-deposited and annealed electrodeposited FeCrNi obtained from platinum and nickel anodes, analysed both on the surface and in-depth of the coating

		Fe (at%)	Cr (at%)	Ni (at%)	O (at%)	C (at%)	N (at%)	H (at%)/metals
*Pt anode* as-deposited	Surface	3.2	2.7	0.2	36.4	56.2	1.3	—
	In-depth	37.8	15.8	11.7	16.8	16.4	1.5	16.0
*Ni anode* as-deposited	Surface	10.2	2.6	3.2	49.7	33.3	1.0	—
	In-depth	35.2	15.5	21.0	22.2	4.2	1.9	12.0
*Pt anode* annealed	In-depth	36.0	23.3	10.3	22.0	6.5	1.9	3.7
*Ni anode* annealed	In-depth	26.5	24.7	14.6	32.6	0.8	0.8	0.1

The unsputtered surfaces of both as-deposited films are rich in oxygen and carbon, with relatively low metal species contributions, except for the *Ni anode* film, where 10.2 at% Fe was measured. The amount of oxygen in-depth of the coating is relatively large regardless of the anode type used (more than 15 at%). The nitrogen concentration is very low and close to the detection sensitivity limit of the XPS instruments (≈1 at%), suggesting that there is no direct incorporation of Cr(iii)–glycine complexes or uncomplexed glycine molecules inside the coating. As expected, the carbon content is more pronounced for the *Pt anode* film (≈16 at%), whereas for the *Ni anode* coating it reaches 4 at%. Interestingly, the ratio of hydrogen to total metal atoms is quite large (above 12 at%), and slightly higher for the *Pt anode* derived sample.

**Fig. 3:**
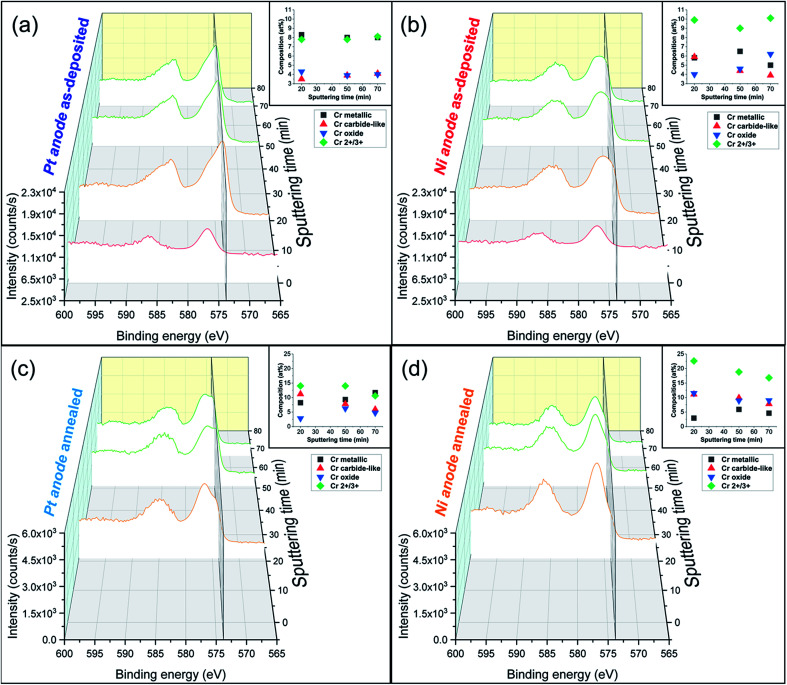
Cr 2p XPS spectra in-depth profiling (0 to 70 min sputtering time) of electrodeposited FeCrNi films with corresponding oxidation states composition graphs: *Pt anode* (a) as-deposited and (b) annealed cases, *Ni anode* (c) as-deposited and (d) annealed ones. Secondary plane at 573.4 eV corresponds to chromium metallic peak.

In contrast, annealed coatings exhibit lower amounts of both C and H in-depth of the film at the expenses of oxygen and chromium, which at% contents increase. Fe and Ni composition variation is thought to be linked with diffusion mechanisms and the elemental transformation of compounds due to the high temperatures involved in the annealing process. Moreover, the composition is not perfectly homogeneous along the thickness of the coatings, especially in the case of the *Ni anode* (variation of Cr peaks intensity and oxidation states in Fig. 3b and d). The carbon content is reduced by approx. three times with respect to the as-deposited counterparts and the hydrogen-to-metals ratio is below 4 at% for the *Pt anode* film and undetectable for the *Ni anode* sample. Thus, it appears that the majority of C and H incorporated within the coatings are weakly bonded and diffuse and outgas upon annealing. Moreover, not only the large carbon concentration, but also the high amount of incorporated hydrogen is responsible for the amorphous character of as-deposited FeCrNi films.

Detailed information about the oxidation states of the different elements from XPS spectra can be found in the ESI.† Overall, the fitting results of both the *Pt anode* and *Ni anode* as-deposited coatings are very similar in terms of binding energies (BE) of the deconvoluted bands. The surface is mainly characterised by carbon and oxygen contribution, with little amounts of chromium oxide and hydroxide (respectively, 2p_3/2_ BEs at 576.2 eV and 577 eV; Fig. 3a surface: 0 min sputtering time), as well as traces of iron in the oxide phase in the *Pt anode* film. Similar results were obtained for the *Ni anode* film (little chromium oxide; Fig. 3b surface: 0 min sputtering time), however, with a more intense iron oxide contribution with respect to the previous case. The elemental evolution from surface to depth shows that Fe and Ni are mainly in metallic state, regardless of anode type. Interestingly, the clear carbon C 1s peak at around 282.5 eV could be associated with chromium carbide (*e.g.* Cr_3_C_2_).^37^ However, it is rather difficult to distinguish between the oxides, carbides and nitrides from the chromium fitting, as their spectral lines are close together. For this reason and as the XRD diffractograms do not show any presence of carbides, it seems more accurate to refer to the fitted peaks as chromium carbide-like (Cr–C) for chromium and carbide-like for carbon. Nevertheless, chromium has a rather constant distribution along the depth of the coating for the *Pt anode* film (Fig. 3a in-depth: 20 to 70 min sputtering time), *i.e.* a larger metallic phase with respect to both oxide and carbide-like contributions. For the *Ni anode* coating (Fig. 3b in-depth: 20 to 70 min sputtering time) the peaks' intensity for Cr metallic is lower compared to the overall oxidation state contribution (Cr^2+/3+^).

For some elements, annealing led to a variation in the oxidation states as a function of the coatings' depth. For the *Ni anode* film, if the nickel content is not affected by thermal treatment (likewise in the *Pt anode* sample), then iron exhibits a rise in the oxide to metallic ratio. After annealing, chromium shows an increase in total contribution (oxide plus carbide-like) with respect to the metallic one (Fig. 3c and d in-depth) for both anode cases. Additionally, when observing other signals (C 1s and O 1s), the thermally treated films show a decrease in carbide-like state in carbon from 12.5 to 6.0 at% for the *Pt anode* coating and from 3.9 to 0.5 at% for the *Ni anode* one. Simultaneously, an increase in Cr oxide contribution in oxygen is observed from 12.4 to 16.7 at% for the *Pt anode* film and from 15.8 to 26.1 at% for the *Ni anode* sample (Table S1 in ESI†). These results show that annealing in general enhances chromium oxide transformation, due to the rather large amount of oxygen present in the coating, especially in the *Ni anode* case.

**Fig. 4:**
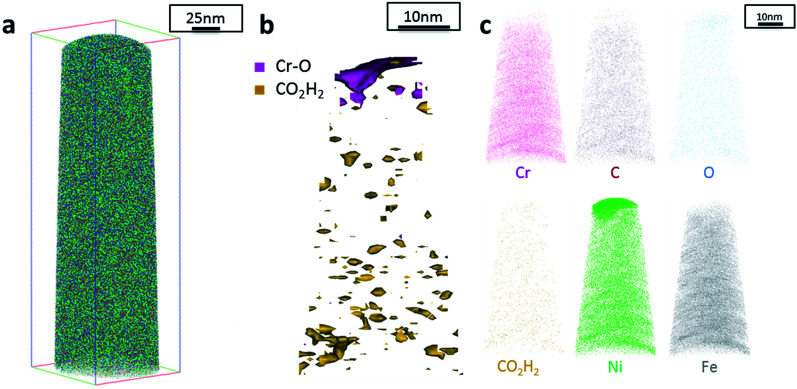
APT results of the FeCrNi tip sample from the *Pt anode* as-deposited coating: (a) 3D elemental reconstruction, (b) isosurface rendering of Cr–O and CO_2_H_2_ compounds and (c) atom maps for different elements/compounds.

**Fig. 5:**
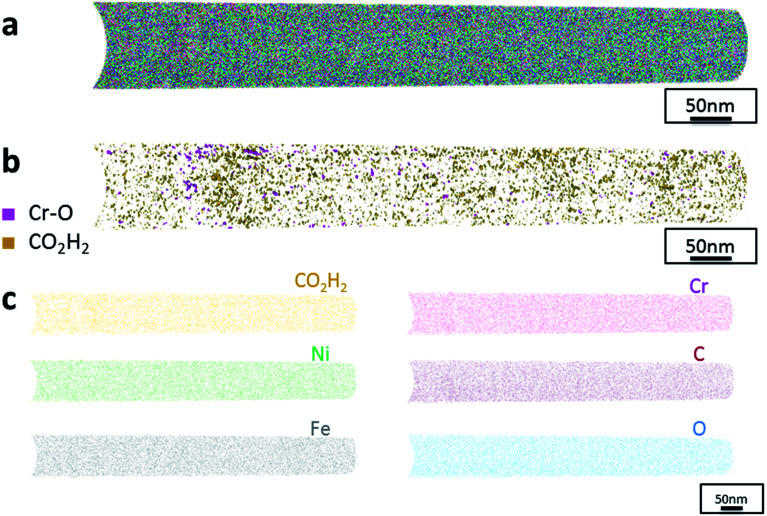
APT results of the FeCrNi tip sample from the *Ni anode* as-deposited coating: (a) 3D elemental reconstruction, (b) isosurface rendering of Cr–O and CO_2_H_2_ compounds and (c) atom maps for different elements/compounds.

Additionally, atom probe tomography (APT) was performed, resulting in 3D elemental reconstructions of nanometre tips for electrodeposited FeCrNi films. Both the as-deposited *Pt anode* (Fig. 4a) and *Ni anode* (Fig. 5a) samples seem to have a similar homogenous elemental distribution. Statistical analysis of the collected data produced some clustering, as depicted in the renderings (Fig. 4b and 5b). The magenta-coloured clusters (30% concentrated isosurfaces) are most probably chromium oxide (termed Cr–O). Except for a large cluster on the top-most part of the *Pt anode* as-deposited case (passive oxide layer at the surface of the film), these Cr–O clusters are infrequent and not uniformly distributed within both case tips. Instead, low concentration (2% isosurfaces) precipitates are heterogeneously distributed within the material (dark-gold colour) corresponding to a mass-over-charge peak identified and labelled as CO_2_H_2_ (more generally a carboxyl molecule). Based on the atom maps (Fig. 4c and 5c), it appears that all the analysed elements have no preferential distribution along the tips (except for the top most part in the *Pt anode* as-deposited film).

**Fig. 6:**
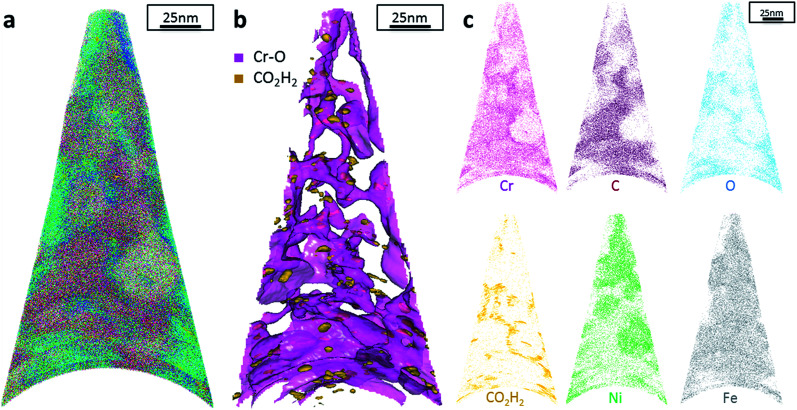
APT results of the FeCrNi tip sample from the *Pt anode* annealed film: (a) 3D elemental reconstruction, (b) isosurface rendering of Cr–O and CO_2_H_2_ compounds and (c) atom maps for different elements/compounds.

**Fig. 7:**
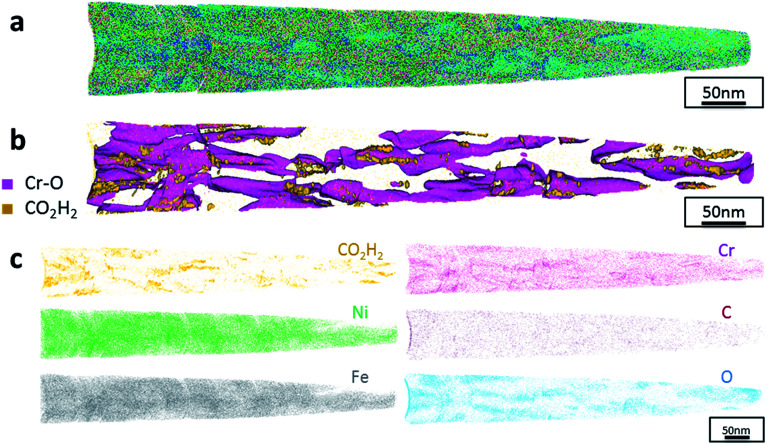
APT results of the FeCrNi tip sample from the *Ni anode* annealed coating: (a) 3D elemental reconstruction, (b) isosurface rendering of Cr–O and CO_2_H_2_ compounds and (c) atom maps for different elements/compounds.

The data from FeCrNi annealed films gives a more complex reconstruction (Fig. 6a and 7a) than from as-deposited ones. Isosurface renderings from clustering analysis (Fig. 6b and 7b) show that Cr–O regions are phase separating (30% concentrated isosurfaces) in a convoluted pattern inside the material. These Cr–O clusters have a chemical microstructure region size of around 20–25 nm. CO_2_H_2_ precipitates are also present in both annealed samples with a slightly higher concentration profile (5% isosurfaces) and are associated with the highly dense Cr–O regions. Atom maps for single elements/compounds (Fig. 6c and 7c) show that some elements are correlated with each other. C and O rich regions follow Cr rich one in the *Pt anode* film. Instead, the annealed *Ni anode* sample has an overall lower carbon concentration with respect to the *Pt anode* counterpart and it seems not to have a preferential spatial arrangement. In both cases, CO_2_H_2_ is present in close proximity to Cr dense areas and at the boundaries of Cr–O clusters. Ni and Fe are not segregating, the former mainly distributed outside of the Cr–O rich zones (mainly for the *Pt anode* annealed coating), whereas the latter is present rather uniformly inside the tips.

### 3.2. Investigation of Cr(iii) complexation

#### 3.2.1. Surface morphology

The surface of the studied samples was mainly semi-bright grey for the *Standard* and *No Cr* electrolytes. Conversely, the coating produced from the *No Cr–Glycine* bath was bright and light-grey.

**Fig. 8:**
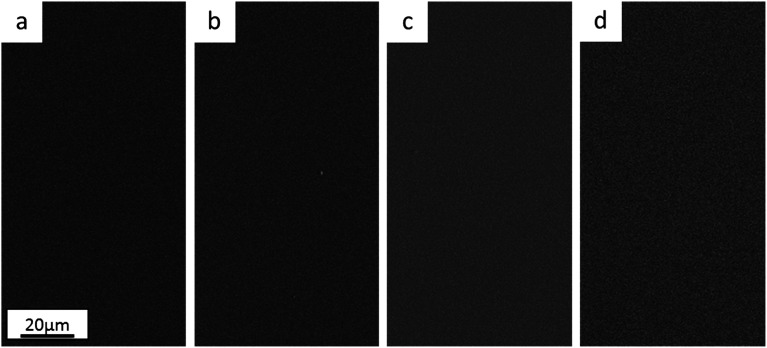
Surface morphology of electrodeposited FeCrNi films from the *Standard* electrolyte with current density of (a) −60 mA cm^−2^ and (b) −80 mA cm^−2^, from the (c) *No Cr–Glycine* bath at −80 mA cm^−2^ and from the (d) *No Cr* bath at −80 mA cm^−2^.

From both optical and scanning electron microscopies, no morphological dissimilarities were observed among the films as depicted in SEM top-view images for the *Standard* (Fig. 8a and b), *No Cr–Glycine* (Fig. 8c), *No Cr* (Fig. 8d) coatings. It should be noted that differently to the coatings described in Section 3.1, (‘Anode role investigation’), no cracks were observed. In fact, in these experiments, a lower total deposited charge was used (*i.e.* lower deposition time). Such behaviour is consistent with the previously mentioned crack formation mechanism.

#### 3.2.2. Crystal structure

**Fig. 9:**
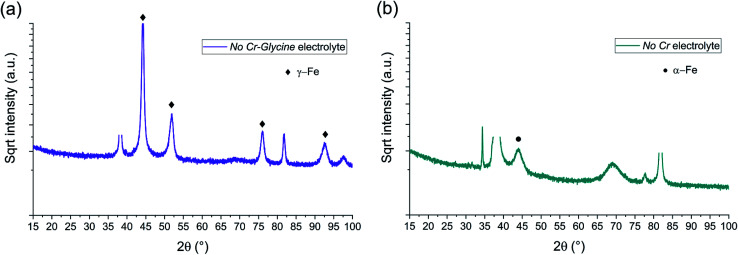
BB-XRD measurements of FeCrNi electrodeposits from the (a) *No Cr–Glycine* and (b) *No Cr* electrolytes.

The crystalline structure studied by means of e*x situ* Bragg–Brentano XRD measurements of FeCrNi electrodeposits is depicted in Fig. 9a for the *No Cr–Glycine* film and in Fig. 9b for the *No Cr* sample. The visible peaks at 2*θ* of 38°, 69° and 82° are linked to fcc Au (111), Si (100) and fcc Au (222) crystal planes (substrate contributions), respectively.

The diffractograms for the *Standard* electrolyte samples are the same as for the as-deposited *Pt anode* coating in Section 3.1: the lack of peaks or a very broad hump at approx. 45° demonstrates that the material is amorphous with a tendency to form α-Fe phases. When neither chromium nor glycine are present in the electrolyte (*No Cr–Glycine* solution), therefore without Cr content in the final coating, the XRD plot (Fig. 9a) clearly shows that the material is γ-Fe (2*θ* peaks at 44°, 52°, 76° and 92°) with an estimated mean crystallite size of 16 nm. Interestingly, the coating obtained from the electrolyte containing glycine and without chromium (*No Cr* bath), shows a crystalline structure similar to ultrafine-grained FeCrNi films (Fig. 9b), with a minor peak at 2*θ* ≈ 45° corresponding to α-Fe phase and a calculated crystallite size of approx. 10 nm.

#### 3.2.3. Elemental composition, oxidation states and speciation

XRF measurements give the Fe–Cr–Ni composition in wt% and current efficiency of the electrodeposited samples depending on the electrolytes: the *Standard* bath samples for −60 and −80 mA cm^−2^ current densities are Fe65–Cr17–Ni18 (C.E. 3.6%) and Fe58–Cr28–Ni14 (C.E. 6.9%), respectively. The *No Cr–Glycine* bath coating is Fe75–Ni25 (C.E. 34.7%) and, the *No Cr* bath film is Fe64–Ni36 (C.E. 3.5%).

The compositions in at% of Fe–Cr–Ni–O–C–N for the studied electrodeposits from various electrolytes were obtained *via* XPS in-depth of the coating (50 min sputtering time) (values listed on Table 3). In the same table, H atomic percentage with respect to the total metal atoms is given from He-ERDA analysis in-depth (20 min sputtering time).

**Table 3:** Elemental composition of Fe–Cr–Ni–O–C–N (XPS) and of H (He-ERDA) of as-deposited FeCrNi electrodeposits obtained from the *Standard*, *No Cr–Glycine* and *No Cr* electrolytes and analysed in-depth

	*i* (mA cm^−2^)	Fe (at%)	Cr (at%)	Ni (at%)	O (at%)	C (at%)	N (at%)	H (at%)/metals
*Standard* electrolyte	−60	40.9	12.7	18.1	16.2	11.2	0.9	13.8
	−80	41.3	17.1	11.9	17.2	11.1	1.4	18.2
*No Cr–Glycine* electrolyte	−80	28.5	0.0	68.8	2.1	0.6	0.0	0.6
*No Cr* electrolyte	−80	42.7	0.0	39.1	15.0	2.6	0.6	10.4

The *Standard* electrolyte coatings show that increasing the current density (therefore the overpotential) from −60 mA cm^−2^ to −80 mA cm^−2^ causes an increase in Cr content from 12.6 at% to 17.1 at%, similarly as measured by XRF. This is in agreement with previous results,^30^ where for high overpotentials, chromium ions are in the mixed mass/charge-transfer region differently to Ni and Fe ions, which are diffusion limited. It should be noted that there are no contents variations for all the non-metallic elements with changes in current density, except for hydrogen, where the ratio to total metal species increased from 13.8 at% to 18.2 at%, suggesting there is a correlation between chromium deposition and hydrogen inclusion. Furthermore, removing both chromium and glycine from the electrolyte (*No Cr–Glycine* bath) cause that no impurities are incorporated in the electrodeposits (C, N and H below 1 at%), oxygen presence is very low (≈2 at%) and that there is more nickel than iron. By removing chromium and keeping glycine in the electrolyte (*No Cr* bath), the composition of the obtained film completely changes: iron content is again higher than nickel, oxygen and hydrogen (≈10 at%) return to high atomic percentages similar to the *Standard* electrolyte coating, and the carbon concentration is no longer negligible (≈3 at%).

From the oxidation point of view, detailed descriptions are present in the ESI.† Nevertheless, the *Standard* electrolyte cases described in this section are identical to the *Pt anode* film described in Section 3.1.3. The increase in current density mainly leads to a rise in the metallic chromium contribution. By comparing No Cr–Glycine and No Cr electrolyte coatings, it could be concluded that the addition of glycine to the bath is linked to an increase in both Fe metallic and oxide contributions, together with a significant decrease in Ni content.

To better understand the possible reactions taking place inside the electrolyte, as well as the speciation/complexation of metal ions obtained from both chemical equilibrium diagrams and UV-vis absorbance spectra, additional literature on acid dissociation and formation/stability constants has been reviewed (see ESI†).

## 4. Discussion

In this work, a comparison among FeCrNi coatings electrodeposited from a Cr(iii)–glycine bath was pursued by using inert platinum and pure nickel anodes. The use of a Ni anode reduces the amount of carbon incorporation, confirming that glycine reactions at the anode are hindered by nickel preferential oxidation to Ni(ii) ions.

In terms of microstructure of as-deposited coatings, the *Pt anode* sample is amorphous whereas the *Ni anode* coating is ultrafine-grained. The three-fold decrease in carbon content in the latter can be related to the amorphisation process, similarly as mentioned in a previous work from our group^26^ on FeCrNi coatings: glycine oxidises at the platinum anode, producing mainly formaldehyde and formic acid, which are sources of carbon atoms incorporated interstitially within the film, leading to an amorphous microstructure. However, the deposition mechanism of both Cr and impurities in that work was not clear.

In the present research, carbon XPS spectra show a peak in the C 1s signal, which can be associated with chromium carbide. However, as the peaks in the Cr 2p signal corresponding to oxides, carbides and nitrides are near each other, it is impossible to completely rule out other possible contributions. A photo-emission study of the interaction of chromium with polymers containing carbonyl groups (*i.e.* C=O) states the formation of Cr–C carbide-like bonds, with a visible peak at 283 eV in the C 1s signal,^38^ similarly as in the outcome of this work. For this reason, carbide-like terminology has been used. Furthermore, the chromium to carbon stoichiometric ratio (Cr : C) was evaluated for the carbide-like fitted contributions (see ESI†). In the as-deposited coatings, the high amount of carbon atoms linked to chromium (*e.g.* Cr : C 1 : 3 for the *Pt anode* sample) is not consistent with the presence of carbides (*i.e.* Cr_3_C_2_, Cr_7_C_3_, Cr_23_C_6_). This fact seems correlated with carbon and carbon moieties (*i.e.* carboxyl groups) being in supersaturated state, probably bonded to Cr and to other molecules such as Cr oxide. After thermal annealing, the Cr : C stoichiometric ratio increases in both anode cases, however, XRD diffractograms and APT results do not show any evidence towards the presence of carbides.

From comparing as-deposited and annealed coatings, it was observed that C and H contents decreased after thermal treatment in all samples. This strengthens the concept that some of the carbon within the coating is linked directly to hydrogen, possibly in a carboxyl manner.

The presence of Fe and Cr oxides after annealing is confirmed by both XRD and XPS. Diffractograms show a low signal-to-noise ratio, associated with possible fluorescence due to the presence of Fe within the coatings. The Fe and Cr oxide phases are most probably present in amorphous state due to the high background at low angles. FeCrNi electrodeposits can be seen as either completely amorphous (as-deposited) or nanocrystalline with an oxide amorphous background (after annealing).

Additionally, from APT measurements, results of as-deposited coatings suggest that all the elements in the film are randomly space-distributed (identical results for different analysed sections of the film), with the exception of carboxyl molecules (labelled as CO_2_H_2_) which seem to be directly incorporated within the film as a precipitate. Depending on the used anode, the two studied coatings have very similar APT outcomes, except for the lower carbon concentration for the *Ni anode* film. APT reconstructions of the annealed samples illustrate more interesting details about the FeCrNi electrodeposits at the nano-scale, which were not possible to observe from microscopic XPS bulk measurements. Cr–O isosurfaces show a complex chromium oxide pattern, which has a chemical region size in the same range as the estimated mean crystallite size using XRD. These Cr–O isosurfaces are associated with carboxyl precipitates (also detected in the as-deposited films). Based on atom maps, there is a clear correlation between C, O and Cr rich regions, as well as Cr–O clusters. In terms of elemental uniformity of the annealed coatings, APT data depict that homogeneity is not maintained at the nano-scale. However, analysis performed at different sections of the same analysed sample always show identical distributions, demonstrating that the annealed coating is reasonably uniform at the micro-scale.

In summary, as a result of anode investigations *via* XRD, XPS and APT, it can be stated that using an inert platinum anode leads to the oxidation of glycine, probably into formaldehyde (CH_2_O), next formic acid (HCOOH), followed by the direct incorporation of a certain amount of carboxyl molecules into the coating. It was observed that it is possible to avoid/decrease glycine-anode reactions by using a nickel anode. This was confirmed by surface morphology variations between the produced coatings. Elemental analyses depict that carbon and carbon-based moieties contents decrease when using a nickel anode. Moreover, heat treatment leads to different processes. Firstly, the diffusion of impurities, *i.e.* carbon, oxygen and carboxyl precipitates, within the deposit (probably directly incorporated during the plating) distribute in close proximity to chromium/chromium oxide rich regions at grain boundaries. Secondly, annealing seems to be also responsible for the outgassing of carboxyl and –H compounds weakly bonded to chromium. However, this does not fully explain the chemical reactions and the mechanisms responsible for the amorphous structure of electrodeposited FeCrNi.

The study of Cr(iii)–glycine complexation shows that both Cr(iii) and glycine electrolyte addition strongly influence the composition variation and microstructural changes of the electrodeposited FeCrNi films. Based on the collected XPS data, it can be seen that an increase in Cr content (*Standard* electrolytes) is linked to higher hydrogen incorporation within the coatings, with no other metal or impurity (C and N) content variations. This demonstrates that the reduction of complexed Cr(iii) is influenced by a hydroxides and/or hydrides deposition process^4,29^ and probably without direct glycine incorporation, due to low nitrogen content. XRD diffractograms show that the samples are amorphous, as expected from the first part of this study. By comparing the two chromium-free baths, it can be observed that the absence of glycine (*No Cr–Glycine* electrolyte) leads to an impurity-free NiFe coating with very low O content and a high current efficiency (C.E. ≈ 35%). The FeNi electrodeposited film obtained from the electrolyte containing glycine (*No Cr* electrolyte) is characterised by a low C.E. (less than 4%), is rich in O and H, but contains a low amount of C. In the first case (NiFe film), the higher amount of Ni is due to a much larger Ni ions concentration within the bath with respect to Fe (Ni : Fe molar ratio > 5), even though iron–nickel is a well-known anomalous co-deposition system. However, the main result is the influence of uncomplexed glycine inside the bath. Glycine did not seem directly responsible for impurity incorporation. However, its presence or, more probably, the presence of its by-products (*i.e.* formaldehyde, formic acid and/or carboxyl group molecules) clearly favours metal hydroxides formation and incorporation. Moreover, competing hydroxides/hydrides reduction processes decrease the efficiency of FeCrNi alloy electrodeposition. XRD results show that without glycine in the bath (NiFe coating without impurities) the film is nanocrystalline, and by adding glycine into the electrolyte (FeNi coating with high hydrogen and oxygen contents and low amount of carbon) the material tends to become amorphous again.

Atomic weights in terms of oxidation state (Table S2 in ESI†) depicts the role of glycine as an intermediator for impurity incorporation. By comparing the resulting coatings from the *No Cr* and *No Cr–Glycine* electrolytes, it can be seen that the addition of glycine significantly increases the Fe metallic contribution, while simultaneously increase the H and O contents within the produced film.

Based on the literature and on the results from speciation/complexation studies (see ESI†), Cr(iii) is complexed with glycine in the FeCrNi *Standard* bath as verified by UV-vis absorbance spectra. Moreover, in the absence of Cr(iii) and glycine in the electrolyte (*No Cr–Glycine* case), Fe and Ni ions are almost certainly following the anomalous co-deposition *via* hydroxides mechanisms.^18,19^ When glycine is added (*No Cr* electrolyte), the most probable and stable glycine complex to be present in the bulk electrolyte is Ni(ii)–glycine (confirmed by chemical equilibrium diagrams). However, due to high local pH near the cathode surface, the presence of carboxylate and hydroxo ions can strongly affect the metals' complexation. Fe(ii)-complex molecules possess a greater tendency to be penetrated due to their lower stability with respect to Ni(ii)–glycine molecules. This explains the increase in Fe deposition with respect to Ni even when the Ni : Fe molar ratio is greater than 5. The amorphous/ultrafine-grained microstructure may be linked to the high hydrogen, oxygen and carbon contents brought about by the co-deposition of carboxylate–hydroxo complexes. Additionally, it seems that when Cr(iii)–glycine complexes are present in the bath (*Standard* case), the correlation between the increase in chromium concentration, as well as hydrogen and carbon contents can only be explained by an incomplete reduction at the cathode. The carboxylate groups are still attached to chromium during the electroreduction process, leading to the presence of metalorganic or organometallic compounds within the coatings.

**Fig. 10:**
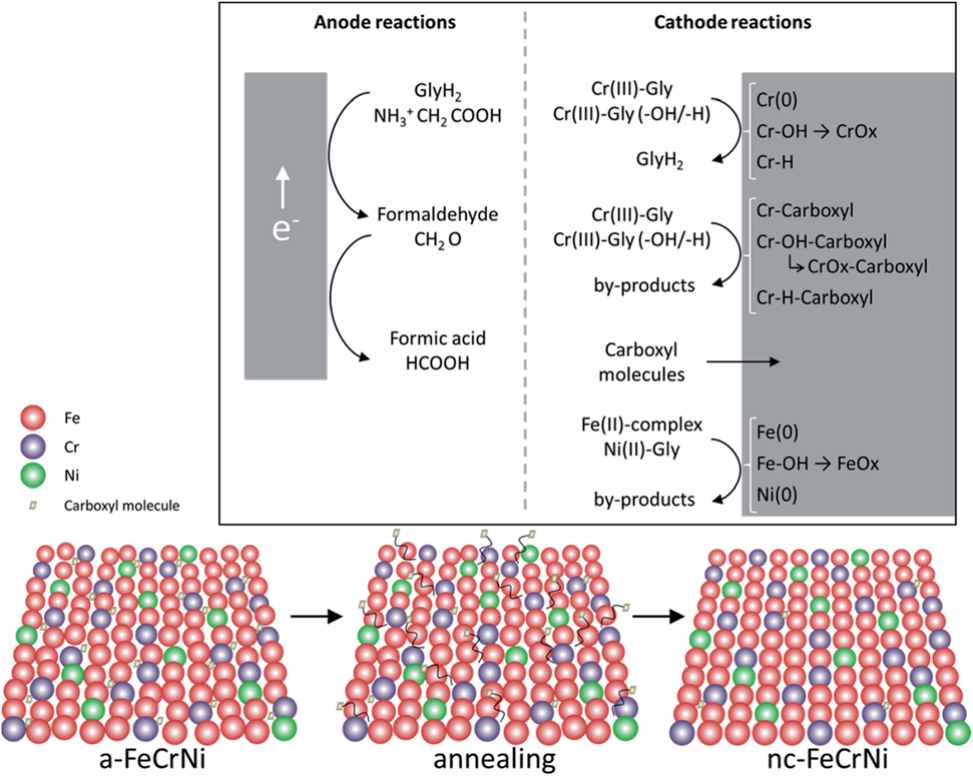
Proposed mechanisms in Cr(iii)–glycine based FeCrNi electrodeposition: (top) anodic/cathodic reactions and (bottom) illustration of microstructural variations due to annealing.

Although further investigations, including *in situ* characterisation, would be necessary to learn more about this system, *ex situ* analysis presented in this work and previously reported information on the studied Cr(iii)–glycine based electrolyte, allow to propose the following reactions (Fig. 10):

- Anode glycine oxidation: in the presence of an inert anode, the oxidation of uncomplexed glycine (first into formaldehyde and later formic acid) becomes relevant.

- Cathodic reactions of Cr(iii):

• Cr(iii)–glycine to metallic chromium: a two-step mechanism with the release of glycine anions. These uncomplexed glycine molecules are free in the electrolyte and can react at the anode or with other metal ions.

• Cr(iii)–glycine hydroxides/hydrides: the presence of strong hydrogen evolution and high local pH at the cathode favours the formation of adsorbed Cr-complex-OH (*via* hydrolysis reaction) and/or Cr-complex-H molecules at the surface.

• Cr(iii)–glycine incomplete deposition: based on annealing results, the drastic decrease in C and H shows that these two elements are weakly bonded to Cr or CrOx (most probably *via* a carboxyl group) within the coating. More precisely, incomplete glycine molecules form weak carbide-like and nitride-like bonds with Cr molecules. High temperatures destabilise both weakly bonded carboxyl and –H group molecules, which are then released from the Cr compound and free to outgas from the film.

- Carboxyl group incorporation: direct inclusion from the electrolyte as precipitate.

- Cathodic reactions of Fe(ii) and Ni(ii): anomalous co-deposition is enhanced due to the high stability of complexed Ni(ii)–glycine and to the fact that Fe(ii) ions have a higher tendency to be complexed by carboxylate/hydroxo ions.

## 5. Conclusions

The electrodeposition of FeCrNi from a ‘green’ Cr(iii)–glycine based electrolyte was investigated. Electroreduction mechanisms were proposed correlating microstructural and composition variations. The anode affects the impurity content and, in turn, the crystallinity. Amorphous and significantly contaminated (high C and H contents) coatings were obtained when using an inert platinum anode, whereas using a nickel anode provided ultrafine-grained films with a lower impurity content. Annealing at 600 °C under an Ar controlled atmosphere produced nanocrystalline γ-Fe coatings (estimated mean crystallite size ≈ 20–25 nm) with substantially decreased carbon and hydrogen contents, showing that these elements are weakly bonded within the material and released during thermal processing. Additional analysis by varying the bath composition depicts that both Cr(iii) and glycine are responsible for the large quantity of impurities. First, glycine oxidises at the anode and forms carboxyl group molecules, such as formic acid. These by-products, together with a local pH increase at the cathode, are the major factors for side-reactions, direct carboxyl moieties inclusion and lower deposition efficiency. Moreover, substantial impurity incorporation (C 11 at% and H 18 at%) in the presence of Cr(iii)–glycine complexes suggests that this electrodeposition process can undergo:

• An incomplete metal–complex reduction, leading to carboxyl molecules weakly bonded to chromium and chromium oxide present within the film,

• Cr–hydroxides/hydrides: formation, adsorption on the cathode's surface and inclusion within the coating.

Impurities from these processes are the main contributor to film amorphisation.

Complexation/speciation studies confirm Cr is complexed with glycine. Additionally, both Ni(ii)–glycine stability and the tendency of Fe(ii) to form carboxyl/hydroxo complexes, enhances anomalous Fe–Ni co-deposition.

Cr(iii)–glycine FeCrNi electroreduction mechanisms were also verified by comparing the as-deposited and thermally treated samples using XPS and APT. The electrodeposits were a mixture of Fe, Cr, Ni, as well as large quantities of Cr oxide, hydrogen, carbon and carbon-based impurities. Films obtained using a nickel anode exhibited lower carbon and carboxyl moieties contents than when using a platinum anode. Further annealing demonstrated that CrO_*x*_ preferentially segregated at the grain boundaries and was associated to carbon-based precipitates.

This study provides guidelines on future electrodeposition of FeCrNi coatings by using a ‘green’ Cr(iii) electrolyte, paving the way for a cost-effective, versatile and non-toxic chromium-based deposition technique, which could be used for many advanced scopes (*e.g.* stainless steel-like FeCrNi micro-nanocomponents for bio-medical applications).

## Conflicts of interest

The authors declare no conflicts of interest.

## Supplementary Material

RA-009-C9RA04262H-s001
