# Supplementary material for: ‘Green’ Cr(iii)–glycine electrolyte for the production of FeCrNi coatings: electrodeposition mechanisms and role of by-products in terms of coating composition and microstructure
Source: RSC Adv. 2019 Aug 16;9(44):25762–75. doi: 10.1039/c9ra04262h (PMC9070400; doi:10.1039/c9ra04262h)
Supplement: RA-009-C9RA04262H-s001 [file RA-009-C9RA04262H-s001.pdf]

Electronic Supplementary Material (ESI) for RSC Advances.
This journal is © The Royal Society of Chemistry 2019

Supporting information for

'Green' Cr(III)-glycine electrolyte for the production of FeCrNi coatings: electrodeposition mechanisms and role of by-products in terms of coating composition and microstructure

Enrico Bertero*[a,b], Cristina V. Manzano[a], Eva Pellicer[c], Jordi Sort[c,d], Robert M. Ulfig[e], Stefano Mischler[b], Johann Michler[a], and Laetitia Philippe[a]

[a]*Empa - Swiss Federal Laboratories for Materials Science and Technology, Laboratory for Mechanics of Materials and Nanostructures, Feuerwerkerstrasse 39, 3602, Thun, Switzerland*

[b]*Ecole Polytechnique Fédérale de Lausanne, Tribology and Interfacial Chemistry Group, Materials Institute Station 12 (SCI-STI-SM), 1015 Lausanne, Switzerland*

[c]*Departament de Física, Facultat de Ciències, Universitat Autònoma de Barcelona, 08193 Bellaterra, Spain*

[d]*Institució Catalana de Recerca i Estudis Avançats (ICREA), Pg. Lluís Companys 23, 08010 Barcelona, Spain*

[e]*CAMECA Instruments Inc., 5470 Nobel Drive, Madison, WI 53711, USA*

*Corresponding author

S1. Anode role investigation

S1.1 Surface morphology

Pictures of the various samples (Fig. S1) show the variation in colour due to the use of different anode material in both as-deposited and annealed cases.

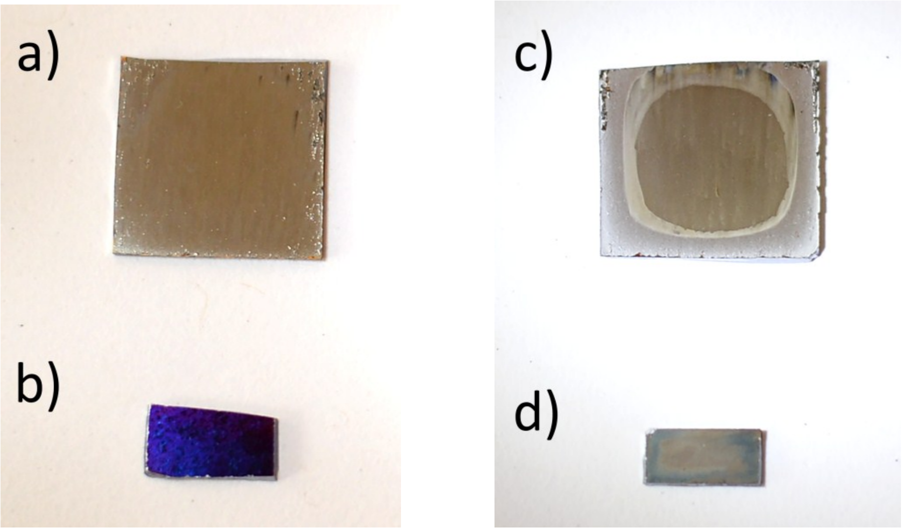

Figure S1. Pictures of electrodeposited FeCrNi coatings: *Pt anode* cases (a) as-deposited and (b) annealed; *Ni anode* cases (c) as-deposited and (d) annealed.

S1.2 X-ray photoelectron spectroscopy (XPS)

The signal from the *Pt anode* and *Ni anode* as-deposited samples are similar, therefore they possess comparable peak fittings.

At surface level, the carbon C 1s signal can be divided into three peaks (Fig. S2a and S3a), i.e. C-C and/or C-H at 284.8 eV, C-OH at 286.4 eV and C=O groups at 288.6 eV. The oxygen O 1s peak at 529.5 eV is attributed to metal oxides. The transition at 531.3 eV may be associated to carboxylic (C-OH, -COOH) and/or hydroxyl (-OH; metal hydroxides) groups, whereas the peak at 532.5 eV may be linked to carbonyl groups (C=O). The spectral components of Fe $2p_{3/2}$ possess binding energies (BEs) of 709.5 eV, 711.7 eV and 714.8 eV. The first and third BE values may be attributed to iron oxide (FeO) and its satellite peaks, while the second transition matches iron in hydroxide form (FeOOH). The chromium Cr $2p_{3/2}$ signal (Cr 2p spectra in Fig. S2a and S3a) can be deconvoluted into two major peaks, i.e. at 576.2 eV and approx. 577 eV corresponding to Cr_2O_3 and $Cr(OH)_3$[1], respectively. Interestingly, only the *Ni anode* FeCrNi coating exhibits a distinguishable Ni $2p_{3/2}$ signal on the surface (figure not shown), attributed to nickel oxide, with two main peaks at 853.4 eV and 856.0 eV and two broad satellites at 860.5 eV and 863.8 eV[2].

In depth of the coating (Fig. S2b and S3b), the O 1s spectral components can be fitted assuming two contributions. The first, located at 529.7 eV, is assigned to chromium oxide, whereas the second at 530.6 eV may be associated with iron oxide. The well-resolved C 1s peak at 282.5 eV may be associated to chromium carbide (Cr_3C_2)[3] or a carbide-like metallic mixture. This is partially confirmed by the Cr $2p_{3/2}$ spectra, in which three peaks fit the signal at BEs of 573.4 eV, 574.6 eV and 576.3 eV, corresponding to metallic chromium, carbide-like bond (Cr-C) and Cr oxide (most probably Cr_2O_3), respectively. However, it is difficult to separate the two contributions as the chromium carbides and oxides are so close to one another. Even though the amount of nitrogen within the coating is minimal, a peak is still registered at 396.7 eV, most probably associated with chromium nitride (CrN)[4] or nitride-like

bond to chromium (Cr-N). In contrast, the chemical state of Fe is mainly metallic with a well-defined peak at 706.5 eV and a small convoluted peak at approx. 711 eV, possibly linked to magnetite (Fe_3O_4). Likewise, Ni $2p_{3/2}$ core-level XPS spectra present a typical metallic peak fitting (BE at 852.1 eV and two satellites at 856.6 eV and 858.6 eV).

After annealing, the samples surfaces were heavily cracked and damaged, probably caused by the occurrence of hydrogen degassing and crack expansion. For this reason, the XPS spectra were only evaluated after sputtering, in-depth of the coating. In the case of the *Pt anode* FeCrNi film, all elements have similar peak fitting as in the as-deposited sample, however, with different intensities (Fig. S2c). The overall oxidation signals (oxide and carbide-like) increased and became comparable to the metallic signals. Here, the contribution of nitrogen is weak, however the BE at 396.2 eV may correspond to a nitride-like bonding. In contrast, the annealed *Ni anode* FeCrNi film shows neither carbon nor nitrogen peaks. The remaining elements (i.e. Fe, Cr, Ni and O) have identical peak fitting as for the as-deposited counterpart, although the metallic chromium signal (Fig. S3c) is rather low in comparison to the enhanced contribution from oxide and carbide-like states.

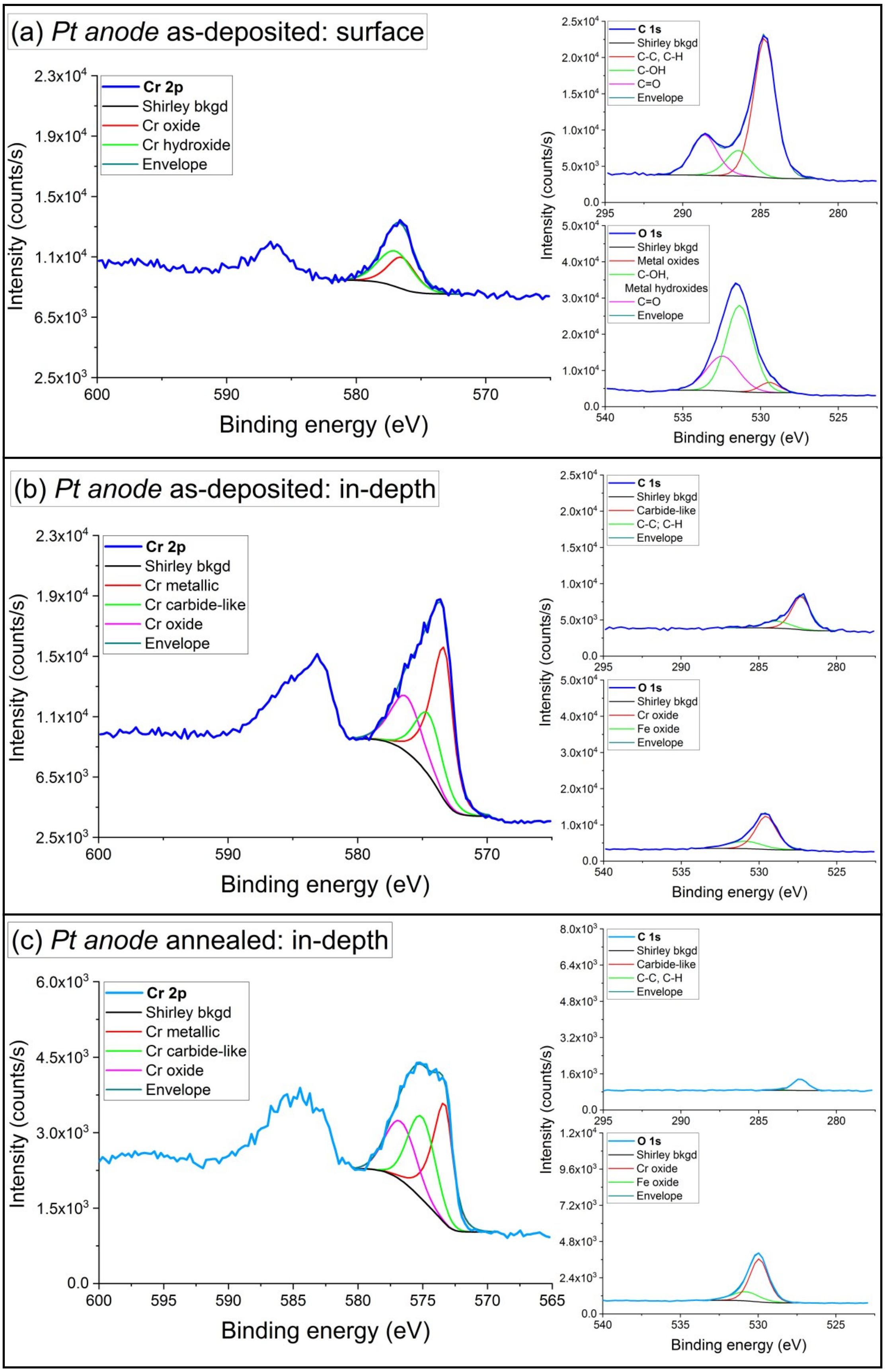

Figure S2. Cr 2p, C 1s and O 1s XPS fitted spectra of electrodeposited *Pt anode* FeCrNi film: (a) surface and (b) in-depth (approx. 50 min sputtering) contributions of the as-deposited sample; (c) in-depth (approx. 50 min sputtering) results of the annealed case.

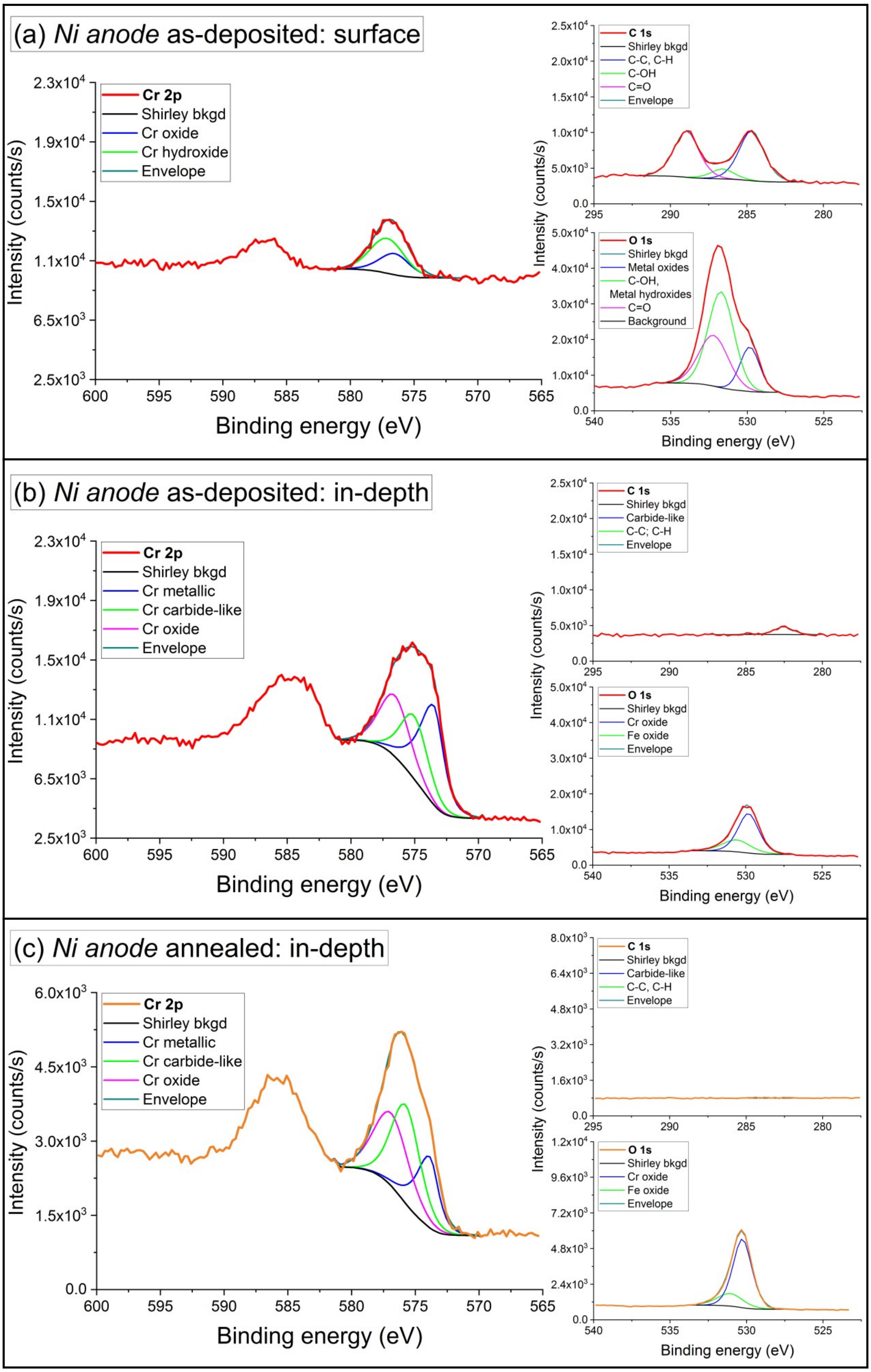

Figure S3. Cr 2p, C 1s and O 1s XPS fitted spectra of electrodeposited *Ni anode* FeCrNi film: (a) surface and (b) in-depth (approx. 50 min sputtering) contributions of the as-deposited sample; (c) in-depth (approx. 50 min sputtering) results of the annealed case.

Table S1 shows the atomic weights for the different elements as a function of their oxidation state, evaluated in-depth of the coating (approx. 50 min sputtering time). The Fe to O stoichiometric ratio is approx. 3 to 4 in all cases, which confirms that the iron oxide is in Fe_3O_4 form. It is difficult to put forward definite conclusions regarding chromium, due to complex peak fitting. For the oxide, in all cases the Cr to O proportion is 1:3, which is in contrast with both fitting of Cr 2p and O 1s, stating chromium oxide is in the Cr_2O_3 form. Instead, chromium to carbon stoichiometric ratio (Cr:C) considering carbide-like contributions, shows much broader variations: *Pt anode* as-deposited (C16.4 at%) Cr:C is 1:3, *Pt anode* annealed (C6.5 at%) Cr:C is 1:1, *Ni anode* as-deposited (C4.2 at%) Cr:C is 1:1 and *Ni anode* annealed (C0.8 at%) Cr:C is 20:1. These differences may also be associated to a partial superimposition of the Cr-C contribution with Cr oxide, making presence of Cr_2O_3 more probable. Nevertheless, the material can still be considered as stainless steel-like FeCrNi, having metallic stoichiometric proportion (Fe:Cr:Ni) similar to austenitic stainless steel.

Table S1. Atomic weight of Fe-Cr-Ni-O-C-N in function of oxidation states from XPS peaks fitting of electrodeposited FeCrNi: *Pt anode* and *Ni anode*, as-deposited and annealed films, analysed in-depth

	Fe (at%)		Cr (at%)		Ni (at%)		O (at%)		C (at%)		N (at%)	
***Pt anode* as-deposited**	Fe met.	34.5	Cr met.	8.0	Ni met.	11.7	Cr oxide	12.4	Carbide-like	12.5	Nitride -like	1.5
	Fe oxide	3.3	Cr-C	3.9			Fe oxide	4.4	C-C, C-H	3.9		
			Cr oxide	3.9								
***Ni anode* as-deposited**	Fe met.	31.5	Cr met.	6.5	Ni met.	21.0	Cr oxide	15.8	Carbide-like	3.9	Nitride -like	1.9
	Fe oxide	3.7	Cr-C	4.4			Fe oxide	6.4	C-C, C-H	0.3		
			Cr oxide	4.6								
***Pt anode* annealed**	Fe met.	32.2	Cr met.	9.3	Ni met.	10.3	Cr oxide	16.7	Carbide-like	6.0	Nitride -like	1.9
	Fe oxide	3.8	Cr-C	7.8			Fe oxide	5.3	C-C, C-H	0.5		
			Cr oxide	6.2								
***Ni anode* annealed**	Fe met.	22.0	Cr met.	5.9	Ni met.	14.6	Cr oxide	26.1	Carbide-like	0.5	Nitride -like	0.8
	Fe oxide	4.5	Cr-C	9.9			Fe oxide	6.5	C-C, C-H	0.3		
			Cr oxide	8.9								

S2. Investigation of Cr(III) complexation

S2.1 X-ray photoelectron spectroscopy (XPS)

In terms of oxidation states from XPS analysis in-depth, the *Standard* electrolyte cases are similar to the previously described *Pt anode* sample. From Table S2, it can be seen that current density amplification only affects metal contributions, mainly chromium, passing from 5.9 to 10.1 at% from lower to higher applied current density and does not affect the carbide and oxide contributions. For the coating obtained from the *No Cr-Glycine* bath, no visible peaks were registered for the carbon and nitrogen signals and the oxygen O 1s spectral component was fitted with a low intensity peak at BE of 530.6 eV, associated to iron oxide (magnetite or hematite). Ni is present in metal form, the Fe 2p spectra with respect to the *Standard* electrolyte sample shows that the metal contribution slightly decreases, whereas the oxide is more pronounced (BE at 711.5 eV). When comparing the *No Cr* and *No Cr-Glycine* electrolyte films, spectra quantifications show that the glycine addition causes an increase in oxygen O 1s peak intensity (linked to Fe oxides) and the carbon C 1s signal once more fits with a carbide peak (BE at 282.7 eV). It was observed that, iron and nickel $2p_{3/2}$ peaks are affected the most by the glycine addition (Table S2), where the first increased (both metallic and oxide contributions) and the second significantly decreased.

Table S2. Atomic weight of Fe-Cr-Ni-O-C-N in function of oxidation states from XPS peaks fitting of electrodeposited FeCrNi: from *Standard*, *No Cr-Glycine* and *No-Cr* electrolytes, analysed in-depth

	Fe (at%)		Cr (at%)		Ni (at%)		O (at%)		C (at%)		N (at%)	
***Standard* (-60 mA/cm²)**	Fe met.<br>Fe oxide	37.1<br>3.8	Cr met.<br>Cr-C<br>Cr oxide	5.9<br>2.8<br>4.0	Ni met.	18.1	Cr oxide<br>Fe oxide	11.3<br>4.9	Carbide-like<br>C-C, C-H	10.8<br>0.4	Nitride-like	0.9
***Standard* (-80 mA/cm²)**	Fe met.<br>Fe oxide	37.3<br>4.0	Cr met.<br>Cr-C<br>Cr oxide	10.1<br>2.8<br>4.2	Ni met.	11.9	Cr oxide<br>Fe oxide	11.3<br>5.9	Carbide-like<br>C-C, C-H	9.2<br>1.9	Nitride-like	1.4
***No Cr-Glycine* (-80 mA/cm²)**	Fe met.<br>Fe oxide	20.8<br>7.7			Ni met.	68.8	Fe oxide	2.1	C-C, C-H	0.6		
***No Cr* (-80 mA/cm²)**	Fe met.<br>Fe oxide	32.9<br>9.8			Ni met.	39.1	Fe oxide	15.0	Carbide<br>C-C, C-H	1.9<br>0.7	N	0.6

S2.2 Complexation study and chemical equilibrium diagrams

When taking into account the bath acidic pH (approx. 1) and the dissociation constants of various molecules, the existing ions, not including metals, could possibly be: ammonium ($NH_4^+ \rightleftharpoons NH_3 + H^+$; $pK_a = 9.25$[5]), sodium (Na^+), chloride ($HCl \rightleftharpoons Cl^- + H^+$; $pK_a = -5.9$[6]), glycine cation $GlyH_2$ ($NH_3^+CH_2COOH \rightleftharpoons NH_2CH_2COOH + H^+$; $pK_a = 2.34$[7]), formaldehyde ($CH_2O \rightleftharpoons CHO^- + H^+$; $pK_a = 13.27$[7]) and formic acid ($HCOOH \rightleftharpoons HCOO^- + H^+$; $pK_a = 3.75$[7]). However, when considering the complex stability constants of metals and electrolytes ligand ions, monoligand Cr(III)-glycine complexation is favoured[8] ($\log\beta = 8.4$[9]) with respect to Fe(II) ($\log\beta = 4.3$[9,10]) and Ni(II) ($\log\beta = 6.2$[9]) glycine complexes due to the bath preparation process[11]. In the presence of formic acid and/or carboxylate ions, these molecules are likely to complex with Ni(II) ($\log\beta = 3.2$[12]) and Fe(II) ($\log\beta = 2.6$[12]), however not with Cr(III) ($\log\beta = 1.9$[13]) as it is already linked to glycine.

In aqueous solution, the studied transition metal ions (i.e. Fe, Cr and Ni) are stable and take the form of hydrate complexes within the considered pH range, unless previously complexed by ligand molecules. However, hydrolysis is a common reaction when pH and/or temperature are increasing: for example, a rise in pH of above 5 at the cathode can lead to the formation of $Ni(OH)^+$ ($\log\beta = 4.1$[14]) and $Fe(OH)^+$ ($\log\beta = 4.5$[14]) hydroxides ions[15]. It is unlikely that Cr(III)-glycine complex will undergo hydrolysis, due to the low stability constant of Cr(III)-hydroxides ($Cr^{3+} \rightleftharpoons Cr(OH)^{2+} + H^+$; $\log\beta = -4.2$[16]), however this Cr(III)-hydroxo equilibrium can be shifted to the right at higher pH values at the cathode.

Chemical equilibrium diagrams extrapolated from Medusa software show that for the *No Cr* electrolyte with an ionic strength (I) of 2.79 (Fig. S4a), Ni-glycine and Fe hydroxides complexes are favoured when pH increases at the cathode surface. However, in case of *No Cr-Glycine* bath with I equal 2.59 (Fig. S4b), as expected, the most probable complexes to form with the increase in pH are nickel and iron hydroxides.

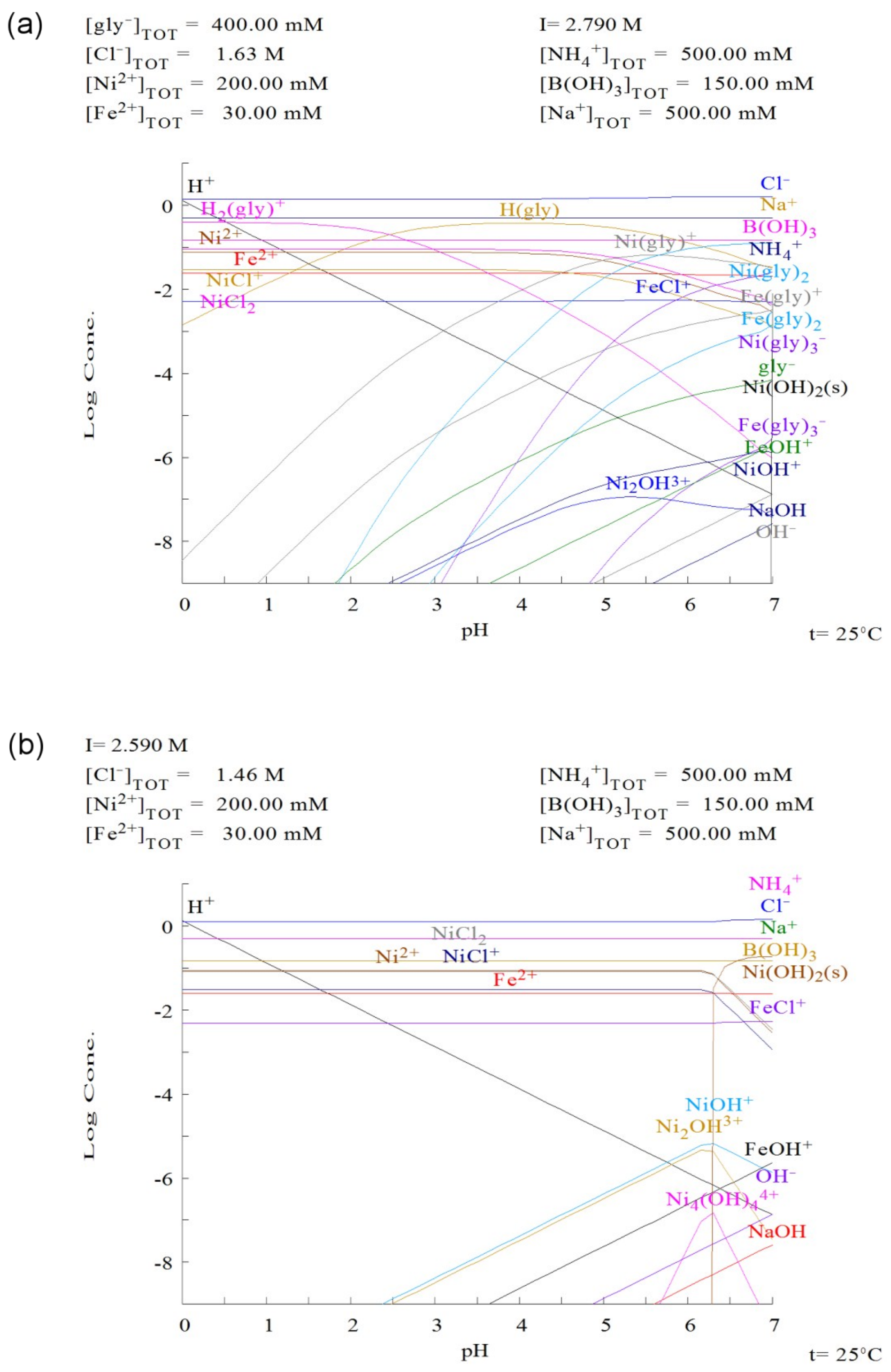

Figure S4. Chemical equilibrium diagrams obtained from Medusa software for (a) *No Cr* and (b) *No Cr-Glycine* electrolytes.

S2.3 UV-vis absorbance spectra

The UV-vis absorption spectra were compared for the FeCrNi electrolytes (both fresh and galvanostatically aged) and the commercial chromium baths (Fig. S5). All FeCrNi electrolytes display similar peaks at wavelengths of λ_1 = 407 nm and λ_2 = 576 nm, corresponding to Cr(III) complexed with glycine[17,18], confirming the results from Section S2.2. A third contribution towards the UV region of the spectra ($\lambda_3 \approx$ 326 nm) seems to appear only after aging the solution using a platinum anode. Fresh commercial decorative trivalent chromium bath exhibits two maxima at 421 and 586 nm, which were identified as Cr(III) complexed with some other organic compounds (e.g. formic acid[19], oxalate[17], polyethylene glycol[20]) and these peaks are similar to the ones from FeCrNi, however, slightly shifted towards longer wavelengths. In comparison, hard hexavalent chromium bath shows a clear peak at 349 nm corresponding to Cr(VI) ions, as observed in other literature works[21–23]. The vicinity of this Cr(VI) contribution with respect to FeCrNi *Pt anode* aged electrolyte local maximum (λ_3) could denote the presence of Cr(VI) ions inside the FeCrNi solution, although the wavelength difference from Cr(VI) and FeCrNi λ_3 maxima is rather large ($\Delta\lambda \approx$ 25 nm). Another explanation is that aging leads to the formation of additional compounds inside the electrolyte, mainly uncomplexed glycine, which can oxidise at the anode to form formic acid or other carboxyl molecules. These compounds could be responsible for the increase in absorbance in the near UV region[24].

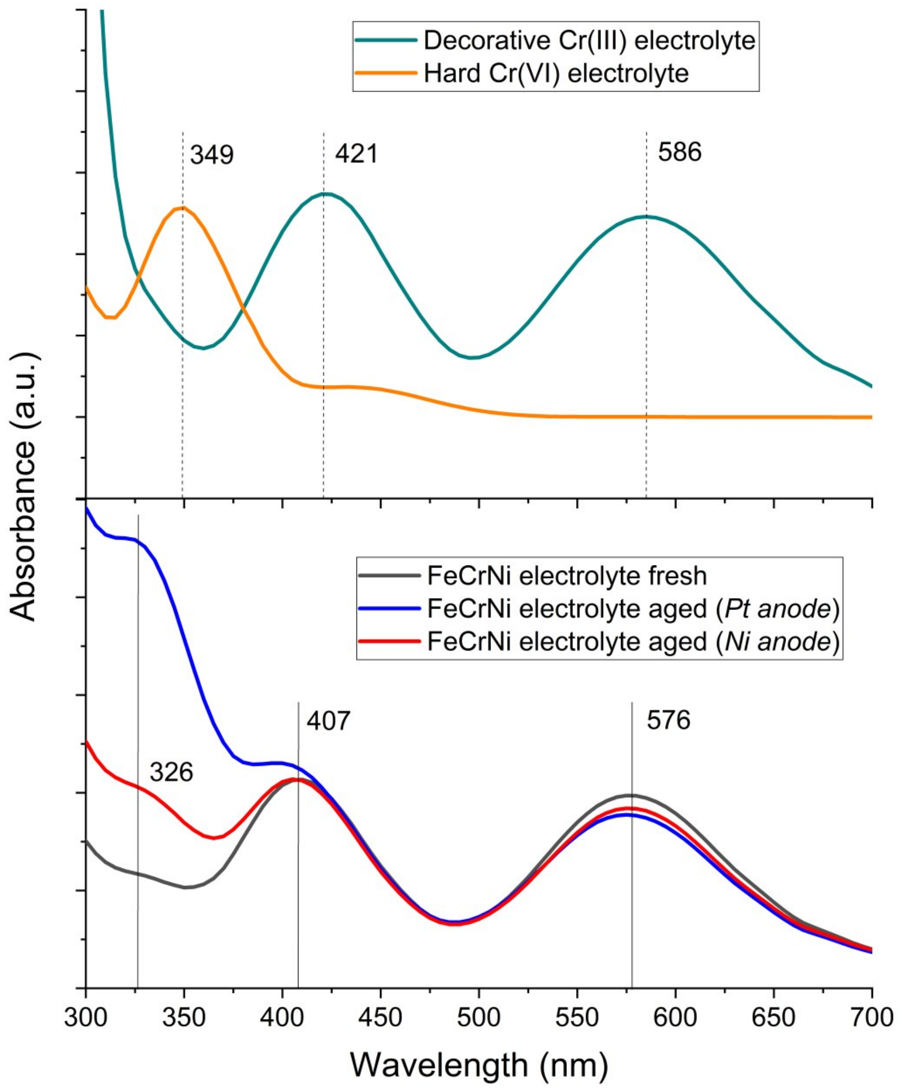

Figure S5. UV-vis absorption spectra of: (bottom) FeCrNi electrolytes and (top) commercial decorative trivalent and hard hexavalent chromium baths.

References

1 K. Asami and K. Hashimoto, *Corros. Sci.*, 1977, **17**, 559–570.

2 N. S. Mcintyre, *Relat. Phenom*, , DOI:10.1021/ac60363a034.

3 K. Petkov, V. Krastev and T. Marinova, *Surf. Interface Anal.*, 1992, **18**, 487–490.

4 O. Nishimura, K. Yabe and M. Iwaki, *J. Electron Spectros. Relat. Phenomena*, 1989, **49**, 335–342.

5 D. D. Perrin, B. Dempsey and E. P. Serjeant, *pKa Prediction for Organic Acids and Bases*, Chapman and Hall, 1981.

6 D. D. Perrin, *Pure Appl. Chem.*, 1969, **20**, 133–236.

7 E. International Union of Pure and Applied Chemistry. Commission on Equilibrium Data., E. P. Serjeant, B. Dempsey and International Union of Pure and Applied Chemistry. Commission on Electrochemical Data., *Ionisation constants of organic acids in aqueous solution*, Pergamon Press, Oxford ;;New York, 1979.

8 J. McDougall, M. El-Sharif and S. Ma, *J. Appl. Electrochem.*, 1998, **28**, 929–934.

9 T. Kiss, I. Sovago and A. Gergely, *Pure Appl. Chem.*, 1991, **63**, 597–638.

10 D. D. Perrin, *J. Chem. Soc.*, 1958, **0**, 3120–3124.

11 M. Hasegawa, S. Yoon, G. Guillonneau, Y. Zhang, C. Frantz, C. Niederberger, A. Weidenkaff, J. Michler and L. Philippe, *Phys. Chem. Chem. Phys.*, 2014, **16**, 26375–84.

12 R. M. Smith and A. E. Martell, *Sci. Total Environ.*, 1987, **64**, 125–147.

13 J. Szynkarczuk, I. Drela and J. Kubicki, *Electrochim. Acta*, 1989, **34**, 399–403.

14 R. M. Smith and A. E. Martell, *Critical Stability Constants*, Springer US, Boston, MA, 1976.

15 W. C. Grande and J. B. Talbot, *J. Electrochem. Soc.*, 1993, **140**, 669–681.

16 N. V Mandich, *Plat. Surf. Finish.*, 1997, **84**, 108–115.

17 S. Surviliene, A. Češūnienė, A. Selskis and R. Butkienė, *Trans. IMF*, 2013, **91**, 24–31.

18 B. Li, A. Lin, X. Wu, Y. Zhang and F. Gan, *J. Alloys Compd.*, 2008, **453**, 93–101.

19 Z. Zeng, Y. Sun and J. Zhang, *Electrochem. commun.*, 2009, **11**, 331–334.

20 N. Van Phuong, S. C. Kwon, J. Y. Lee, J. H. Lee and K. H. Lee, *Surf. Coatings Technol.*, 2012, **206**, 4349–4355.

21 M.-C. Fournier-Salaün and P. Salaün, *CEJC*, 2007, **5**, 1084–1093.

22 S. Goeringer, N. R. de Tacconi, C. R. Chenthamarakshan and K. Rajeshwar, *J. Appl. Electrochem.*, 2000, **30**, 891–897.

23 M. Celebi, M. Yurderi, A. Bulut, M. Kaya and M. Zahmakiran, *Appl. Catal. B Environ.*, 2016, **180**, 53–64.

24 Y. Gao and Y. Zhang, *RSC Adv.*, 2018, **8**, 20719–20725.
